# Supplementary material for: Polyketide Derivatives from the Endophytic Fungus Phaeosphaeria sp. LF5 Isolated from Huperzia serrata and Their Acetylcholinesterase Inhibitory Activities
Source: J Fungi (Basel). 2022 Feb 26;8(3):232. doi: 10.3390/jof8030232 (PMC8955197; doi:10.3390/jof8030232)
Supplement: Supplementary file 1 [file jof-08-00232-s001.zip › jof-1604364-supplementary.pdf]

Supporting Information for

Polyketide Derivatives from the Endophytic Fungus *Phaeosphaeria* sp. LF5 Isolated from *Huperzia serrata* and Their Acetylcholinesterase Inhibitory Activities

Yiwen Xiao [1,2,†], Weizhong Liang [2,†], Zhibin Zhang [1], Ya Wang [2], Shanshan Zhang [2], Jiantao Liu [2], Jun Chang [2], Changjiu Ji [3] and Du Zhu [1,2,*]

1 Key Laboratory of Protection and Utilization of Subtropic Plant Resources of Jiangxi Province, College of Life Sciences, Jiangxi Normal University, Nanchang 330022, China; xyw1152858687@163.com (Y.X.); zzbbio@jxnu.edu.cn (Z.Z.)

2 Key Laboratory of Bioprocess Engineering of Jiangxi Province, College of Life Sciences, Jiangxi Science and Technology Normal University, Nanchang 330013, China; lwz271508312@163.com (W.L.); jxwangya@126.com (Y.W.); zss18135437140@163.com (S.Z.); liujt@jxstnu.edu.cn (J.L.); changjun@jxstnu.edu.cn (J.C.)

3 College of Chemistry and Biological Engineering, Yichun University, Yichun 336000, China; jichjiu999@jxycu.edu.cn

* Correspondence: zhudu@jxstnu.edu.cn; Tel.: +86-79188121934

† These authors contributed equally to this work.

Table S1. Acetylcholinesterase inhibitory activities of the crude extracts
Figure list: HR-ESI-MS, 1D and 2D NMR spectra data for compounds **1-11**
Figure S1. ^{1}H NMR spectrum of compound **1** in CD_3OD
Figure S2. ^{13}C NMR spectrum of compound **1** in CD_3OD
Figure S3. ^{1}H NMR spectrum of compound **2** in CD_3OD
Figure S4. ^{13}C NMR spectrum of compound **2** in CD_3OD
Figure S5. ^{1}H NMR spectrum of compound **3** in CD_3OD
Figure S6. ^{13}C NMR spectrum of compound **3** in CD_3OD
Figure S7. ^{1}H NMR spectrum of compound **4** in CD_3OD
Figure S8. ^{13}C NMR spectrum of compound **4** in CD_3OD
Figure S9. ^{1}H-^{1}H COSY spectrum of compound **4** in CD_3OD
Figure S10. DEPT 90 spectrum of compound **4** in CD_3OD
Figure S11. DEPT 135 spectrum of compound **4** in CD_3OD
Figure S12. HSQC spectrum of compound **4** in CD_3OD
Figure S13. HMBC spectrum of compound **4** in CD_3OD
Figure S14. NOESY spectrum of compound **4** in CD_3OD
Figure S15. HRESIMS spectrum of compound **4**
Figure S16. ^{1}H NMR spectrum of compound **5** in CD_3OD
Figure S17. ^{13}C NMR spectrum of compound **5** in CD_3OD
Figure S18. ^{1}H NMR spectrum of compound **6** in CD_3OD
Figure S19. ^{13}C NMR spectrum of compound **6** in CD_3OD
Figure S20. ^{1}H NMR spectrum of compound **7** in CD_3OD
Figure S21. ^{13}C NMR spectrum of compound **7** in CD_3OD
Figure S22. ^{1}H-^{1}H COSY spectrum of compound **7** in CD_3OD
Figure S23. HSQC spectrum of compound **7** in CD_3OD
Figure S24. HMBC spectrum of compound **7** in CD_3OD
Figure S25. NOESY spectrum of compound **7** in CD_3OD
Figure S26. HRESIMS spectrum of compound **7**
Figure S27. ^{1}H NMR spectrum of compound **8** in CD_3OD
Figure S28. ^{13}C NMR spectrum of compound **8** in CD_3OD
Figure S29. ^{1}H NMR spectrum of compound **9** in DMSO-d_6
Figure S30. ^{13}C NMR spectrum of compound **9** in DMSO-d_6
Figure S31. DEPT 90 spectrum of compound **9** in DMSO-d_6
Figure S32. DEPT 135 spectrum of compound **9** in DMSO-d_6
Figure S33. ^{1}H-^{1}H COSY spectrum of compound **9** in DMSO-d_6
Figure S34. HSQC spectrum of compound **9** in DMSO-d_6
Figure S35. HMBC spectrum of compound **9** in DMSO-d_6
Figure S36. NOESY spectrum of compound **9** in DMSO-d_6
Figure S37. HRESIMS spectrum of compound **9**
Figure S38. ^{1}H NMR spectrum of compound **10** in CD_3OD
Figure S39. ^{13}C NMR spectrum of compound **10** in CD_3OD
Figure S40. ^{1}H NMR spectrum of compound **11** in CD_3OD
Figure S41. ^{13}C NMR spectrum of compound **11** in CD_3OD

Table S1. Acetylcholinesterase inhibitory activities of the crude extracts.

Sample	Inhibition effects (%)[a]	Sample	Inhibition effects (%)[a]
Petroleum ether extract	47.23 ± 0.40	Ethyl acetate extract	82.68 ± 1.60
Hup-A	93.20±0.30	Buthanol extract	15.82 ± 1.20

[a] Expressed as the mean ± SD of three parallel measurements ($p<0.05$).

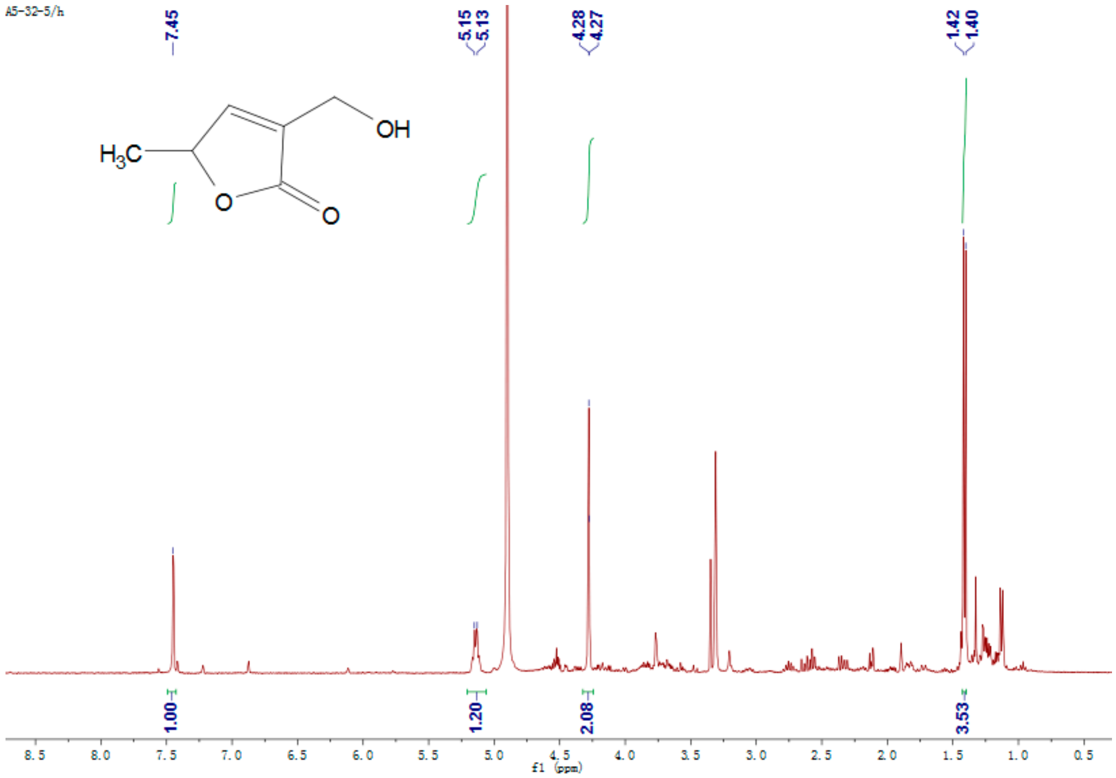

Figure S1. ^{1}H NMR spectrum of compound **1** in CD_3OD.

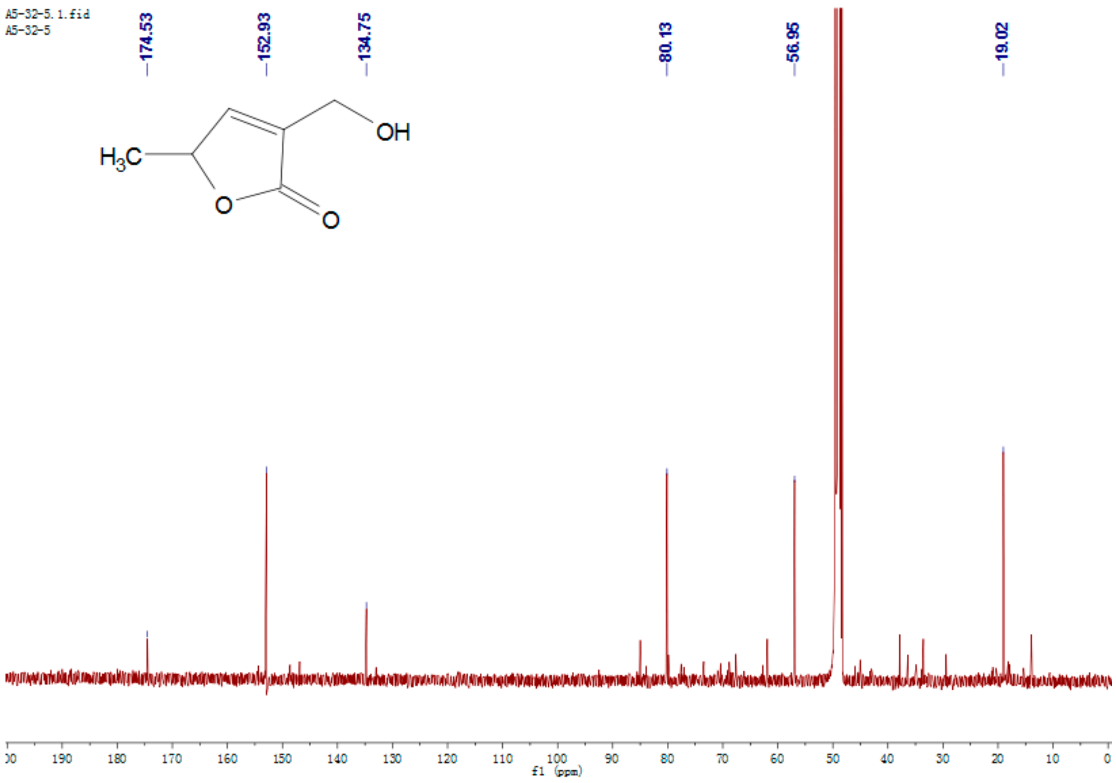

Figure S2. ^{13}C NMR spectrum of compound **1** in CD_3OD.

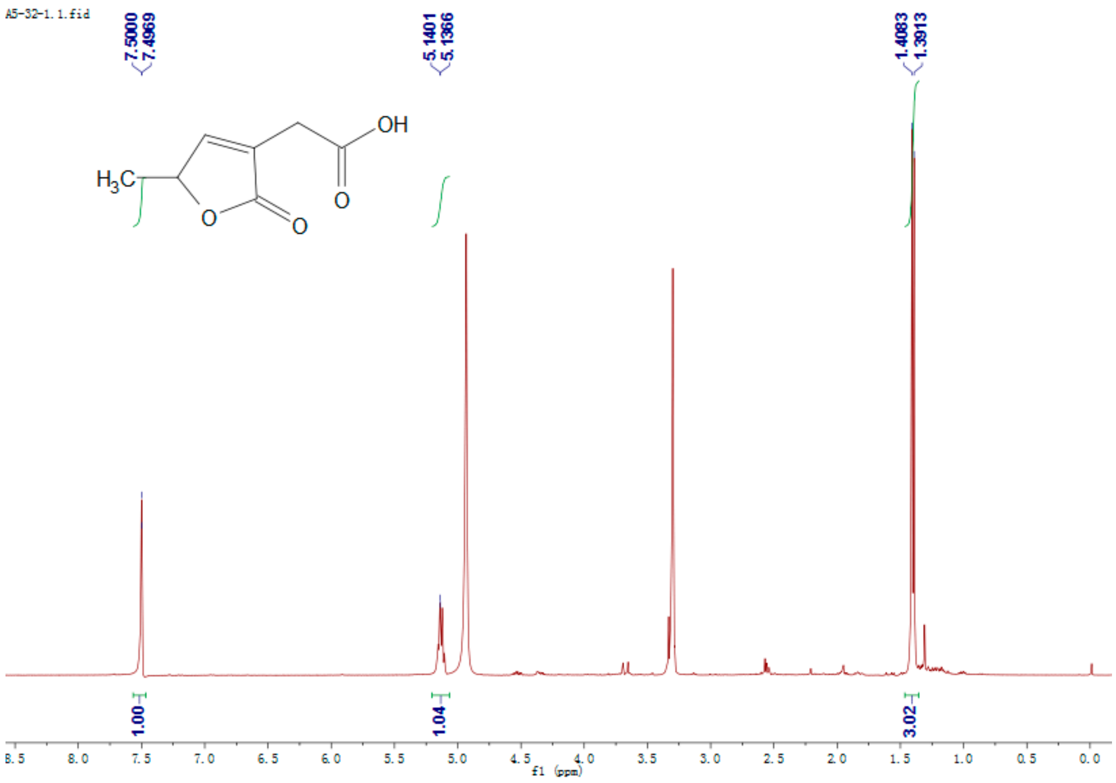

Figure S3. ^{1}H NMR spectrum of compound **2** in CD_3OD.

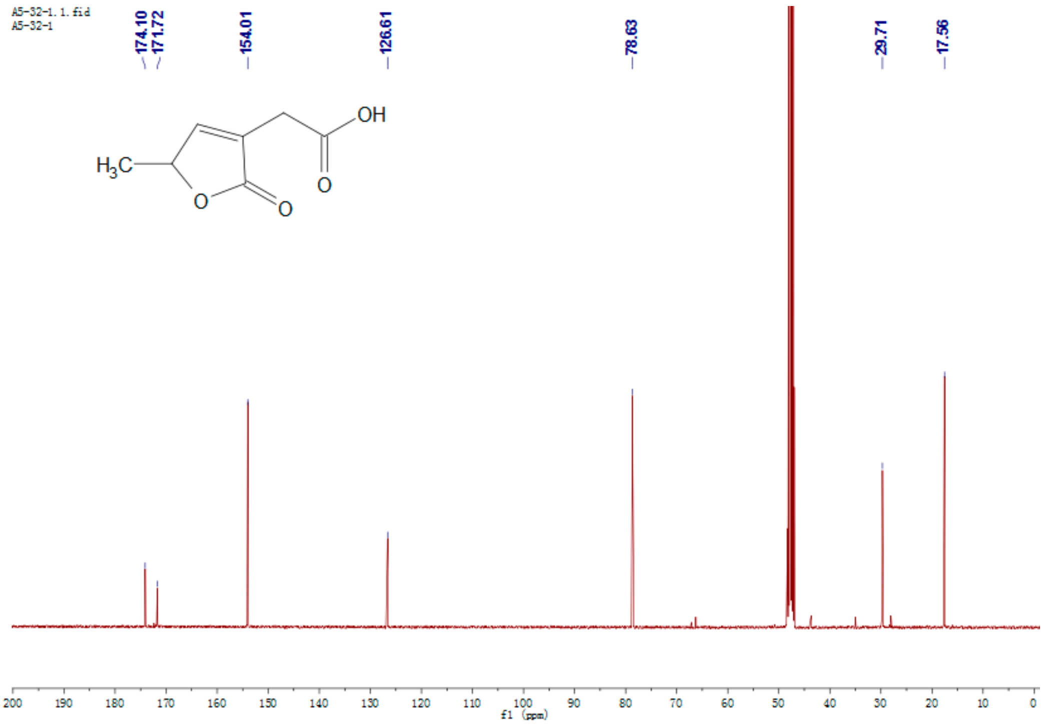

Figure S4. ^{13}C NMR spectrum of compound **2** in CD_3OD.

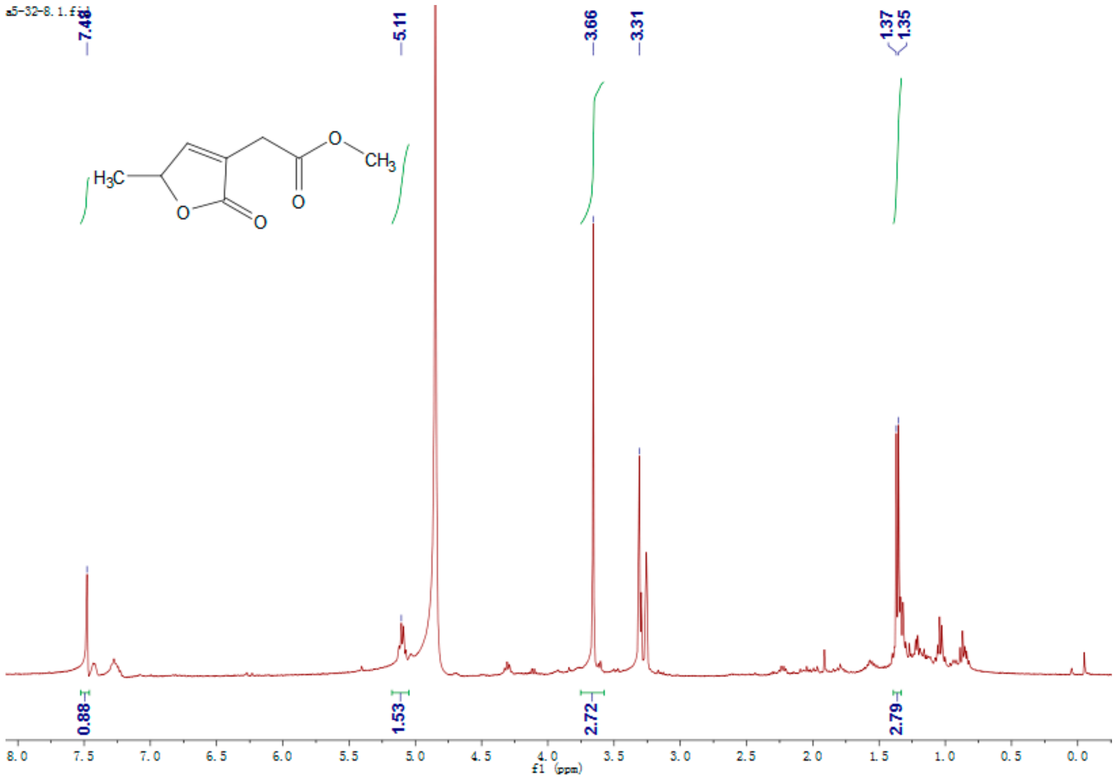

Figure S5. ^{1}H NMR spectrum of compound **3** in CD_3OD.

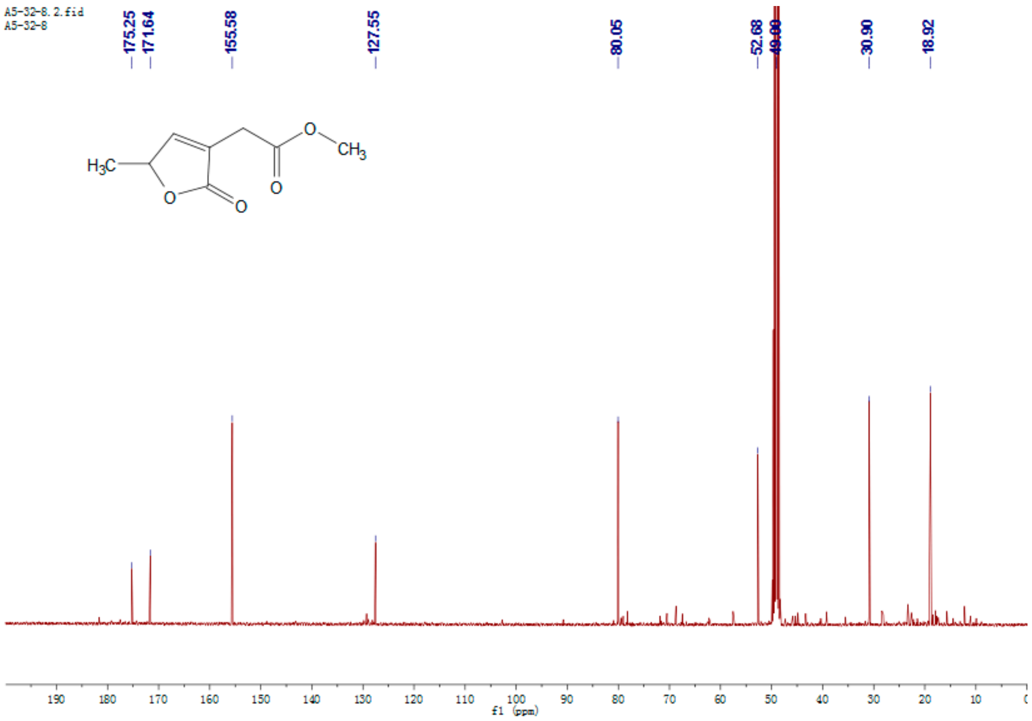

Figure S6. ^{13}C NMR spectrum of compound **3** in CD_3OD.

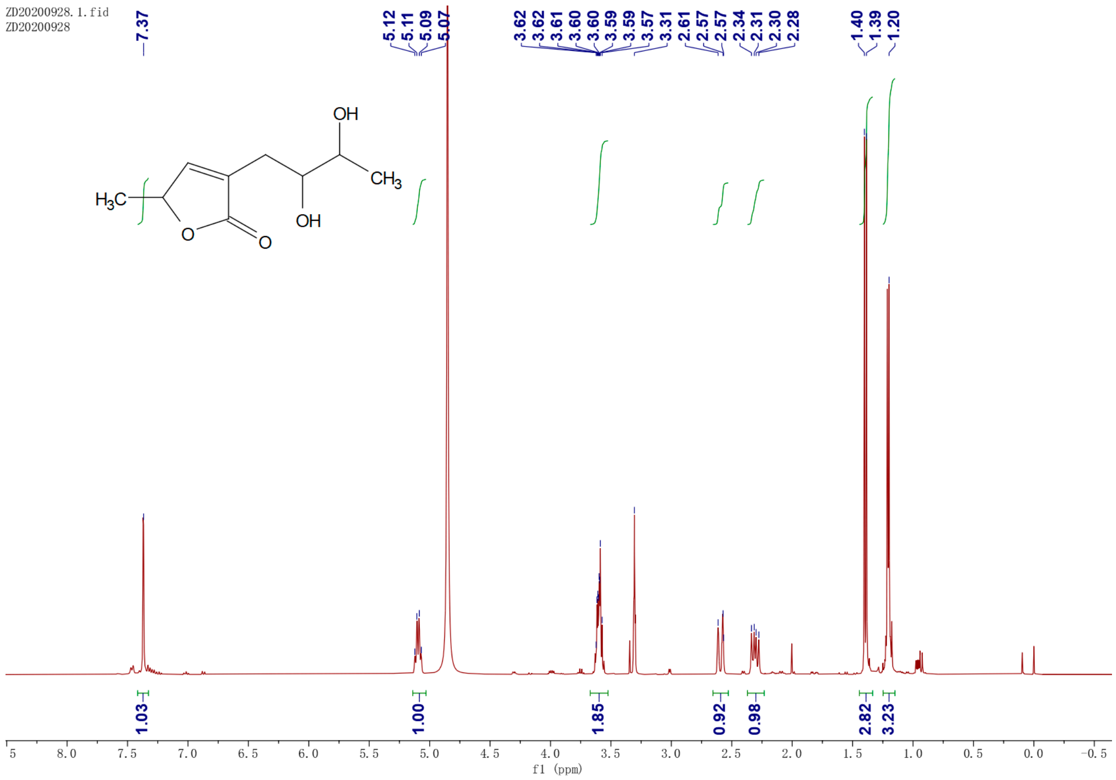

Figure S7. ^{1}H NMR spectrum of compound **4** in CD_3OD.

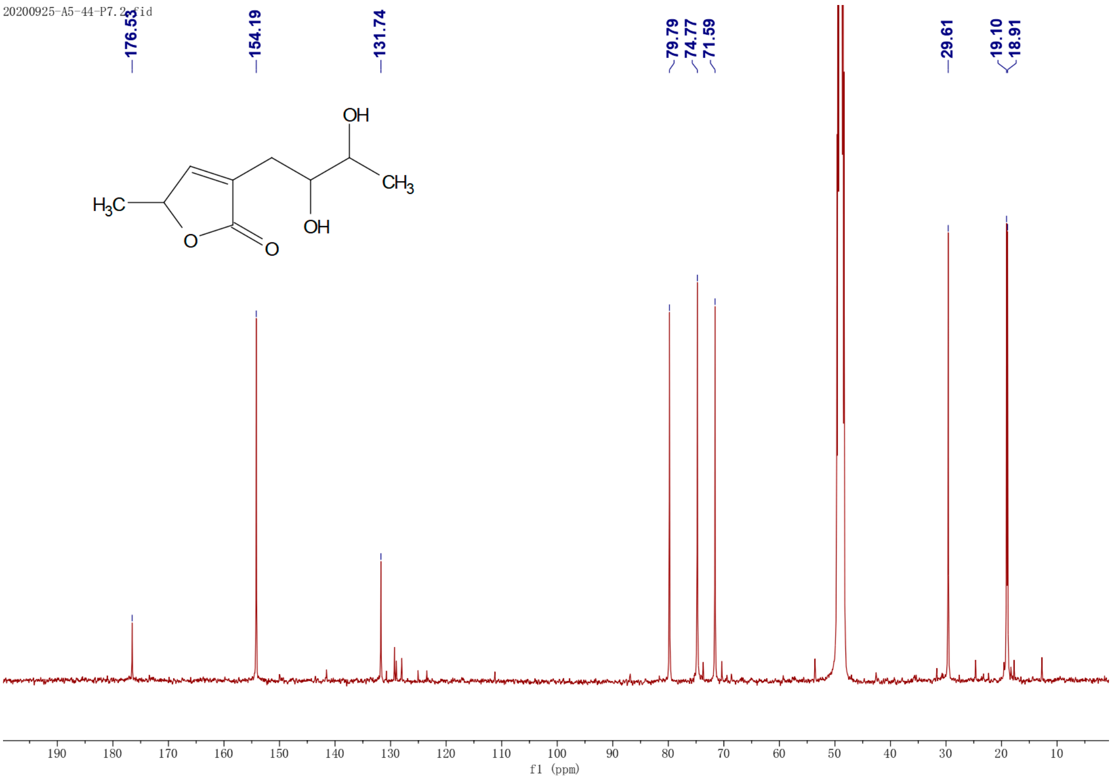

Figure S8. ^{13}C NMR spectrum of compound **4** in CD_3OD.

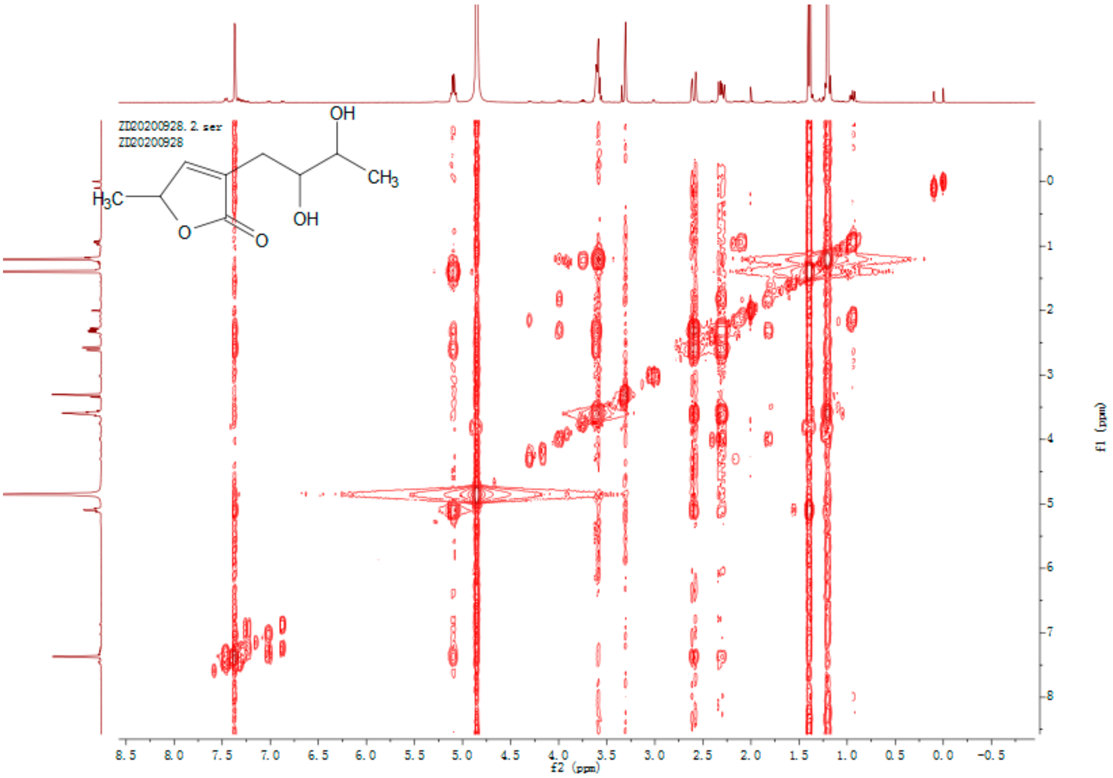

Figure S9. ^{1}H-^{1}H COSY spectrum of compound **4** in CD_3OD.

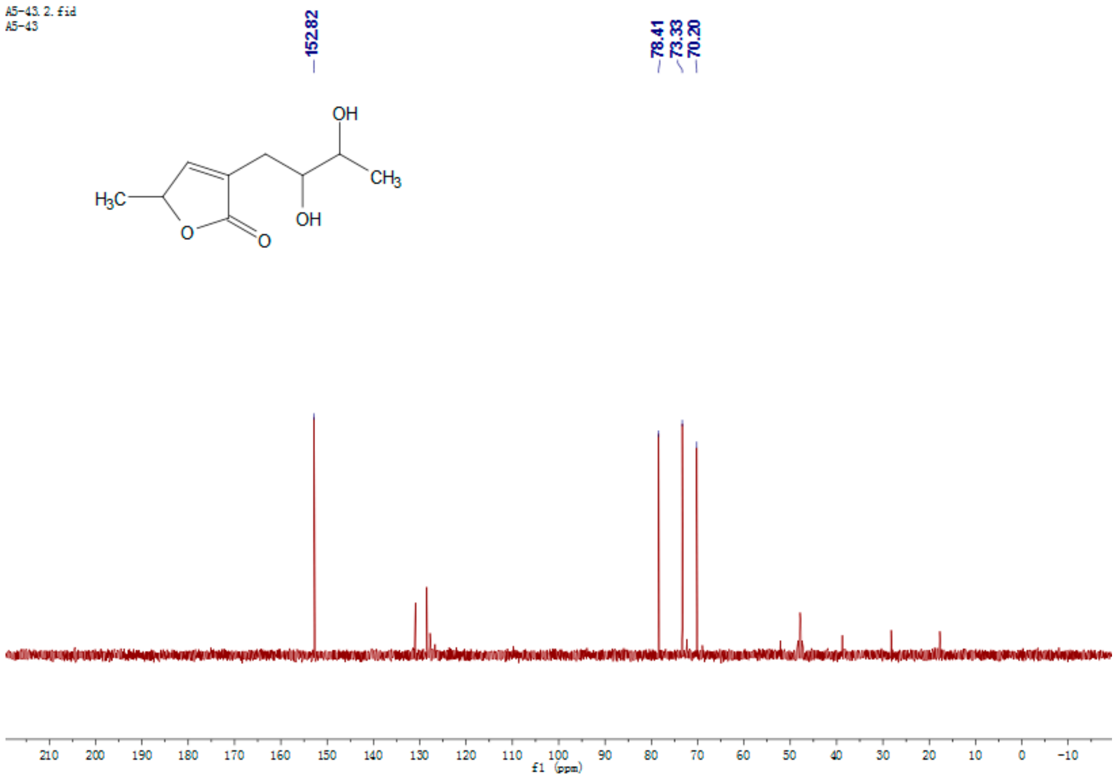

Figure S10. DEPT 90 spectrum of compound **4** in CD_3OD.

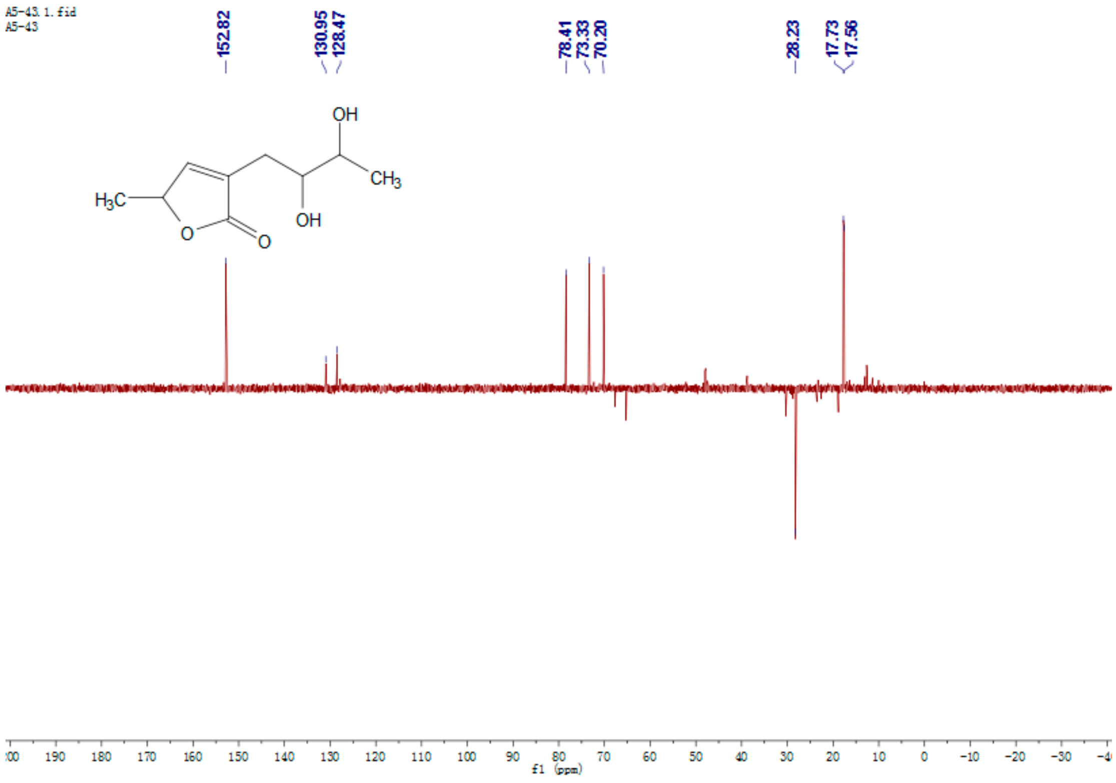

Figure S11. DEPT 135 spectrum of compound **4** in CD_3OD.

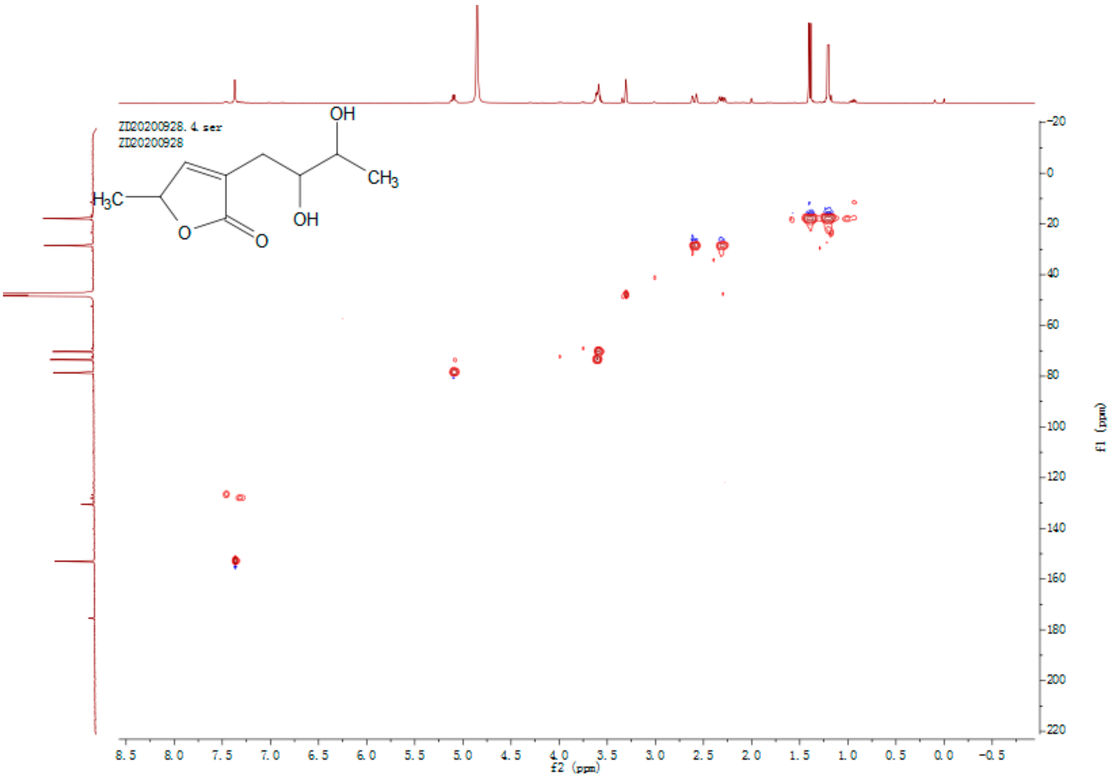

Figure S12. HSQC spectrum of compound **4** in CD_3OD.

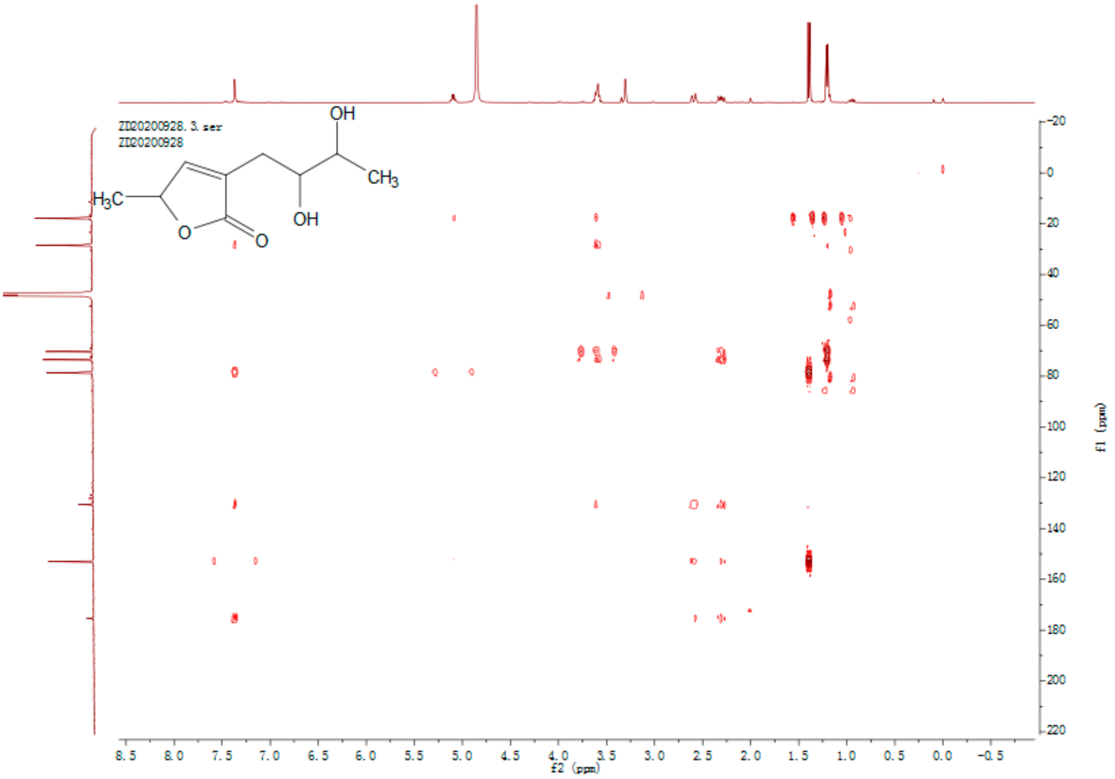

Figure S13. HMBC spectrum of compound **4** in CD_3OD.

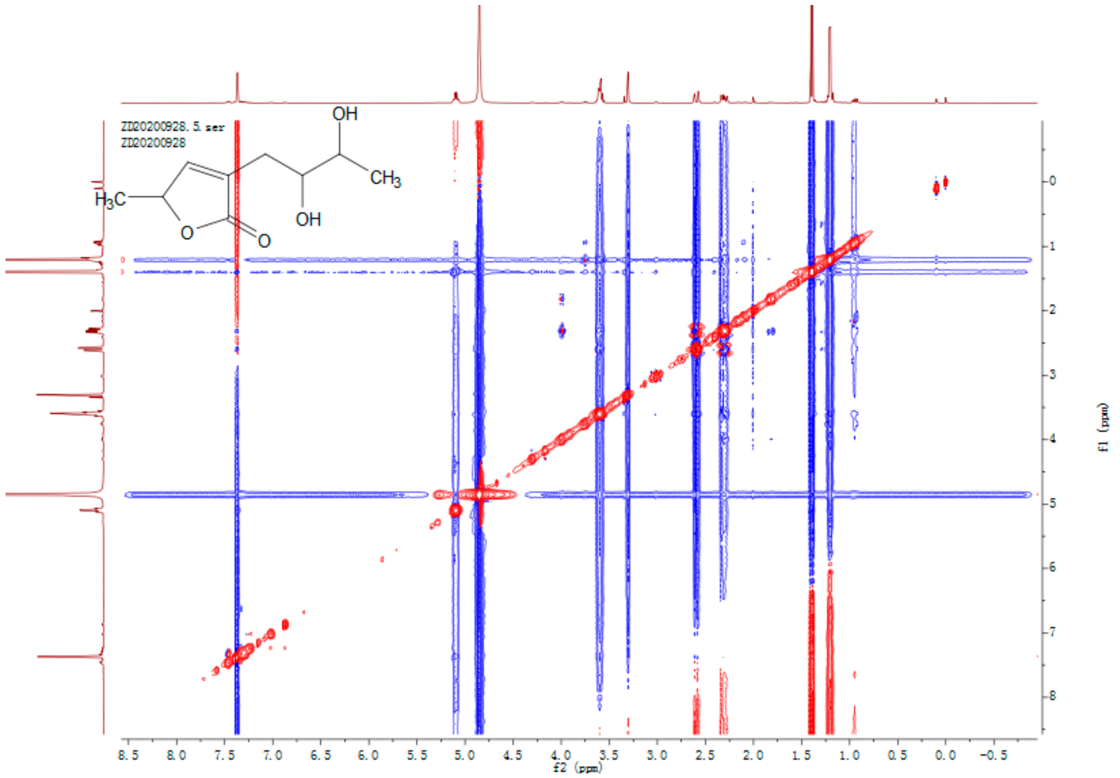

Figure S14. NOESY spectrum of compound **4** in CD_3OD.

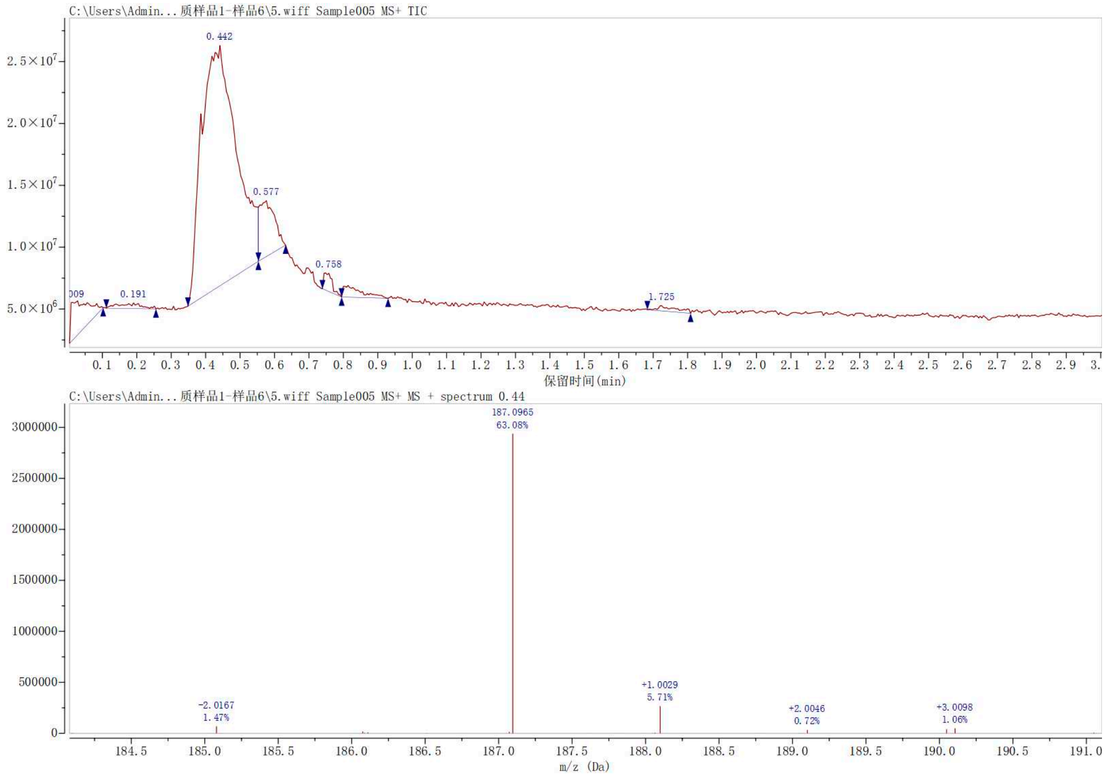

Figure S15. HRESIMS spectrum of compound **4.**

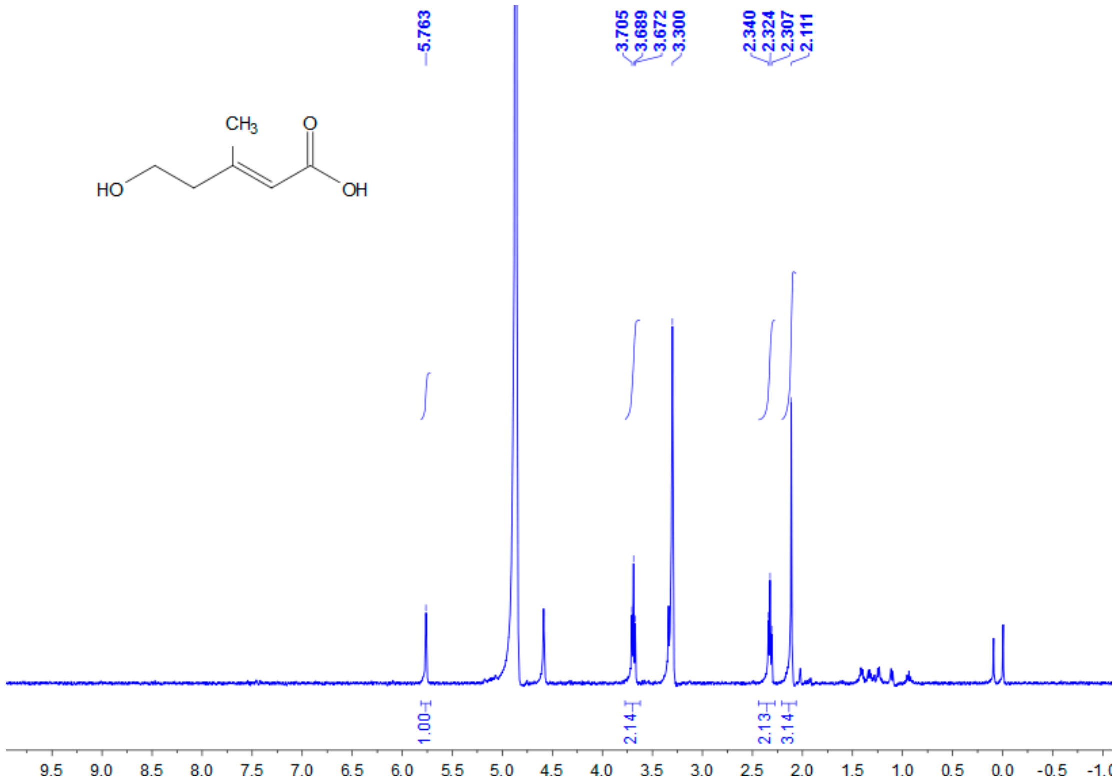

Figure S16. ¹H NMR spectrum of compound **5** in CD_3OD.

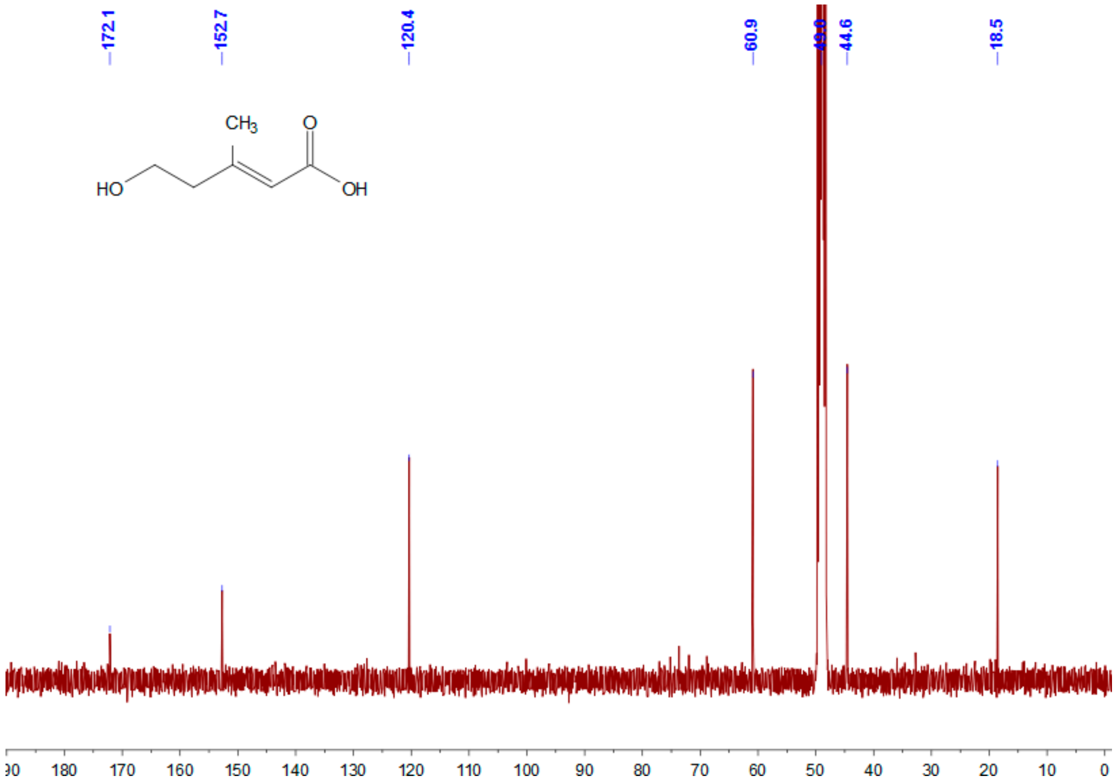

Figure S17. ¹³C NMR spectrum of compound **5** in CD_3OD.

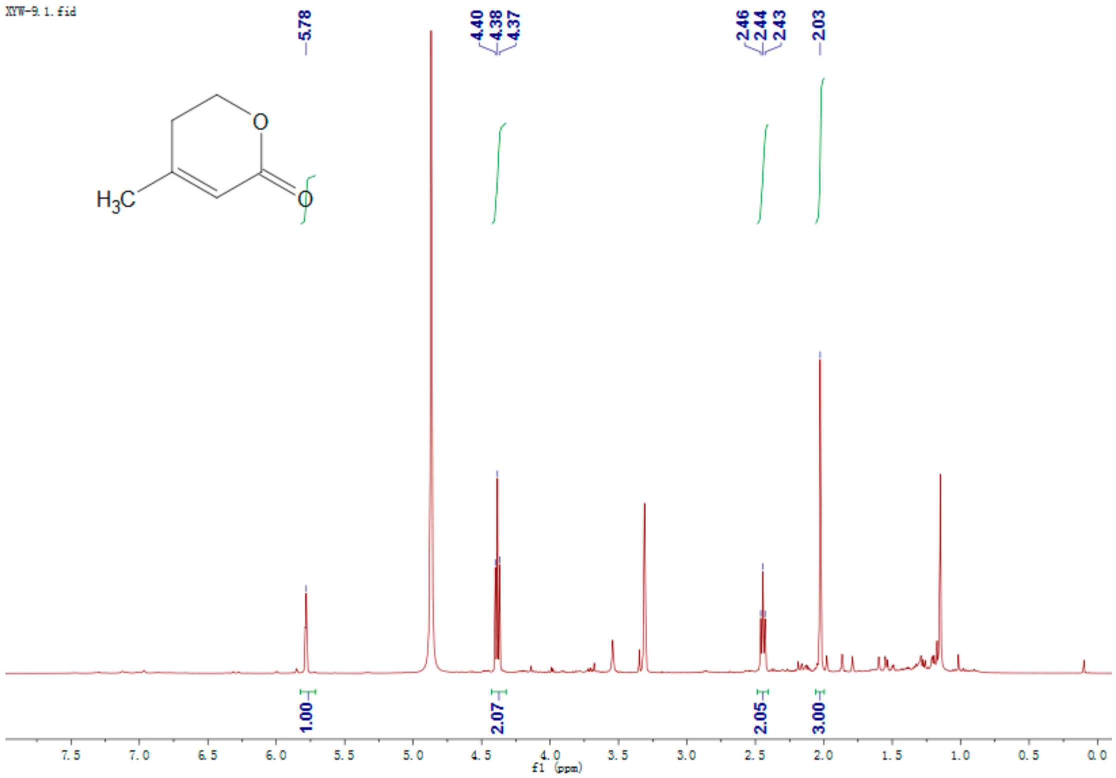

Figure S18. ^{1}H NMR spectrum of compound **6** in CD_3OD.

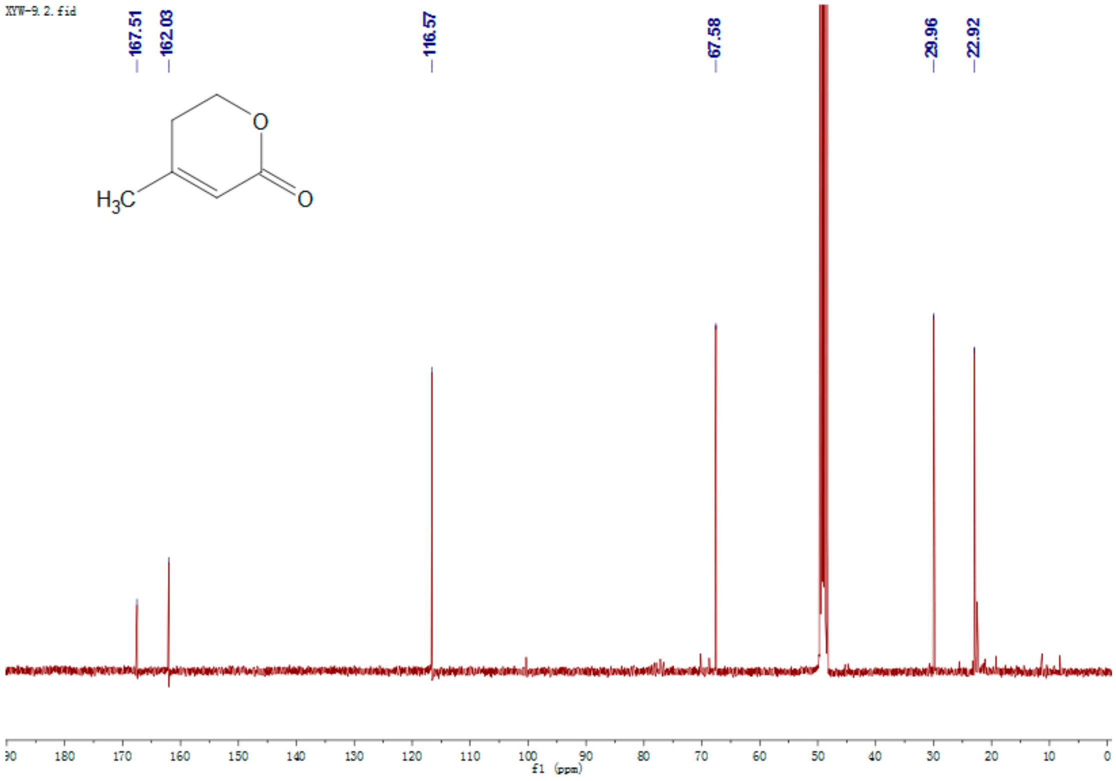

Figure S19. ^{13}C NMR spectrum of compound **6** in CD_3OD.

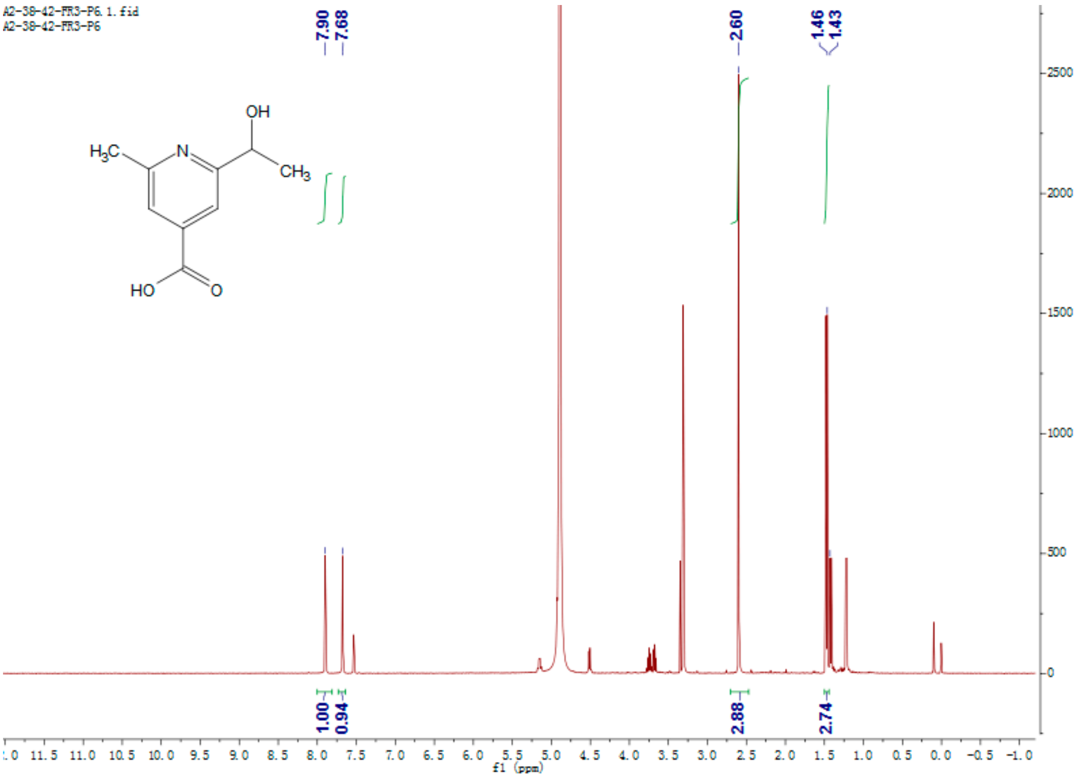

Figure S20. ^{1}H NMR spectrum of compound **7** in CD_3OD.

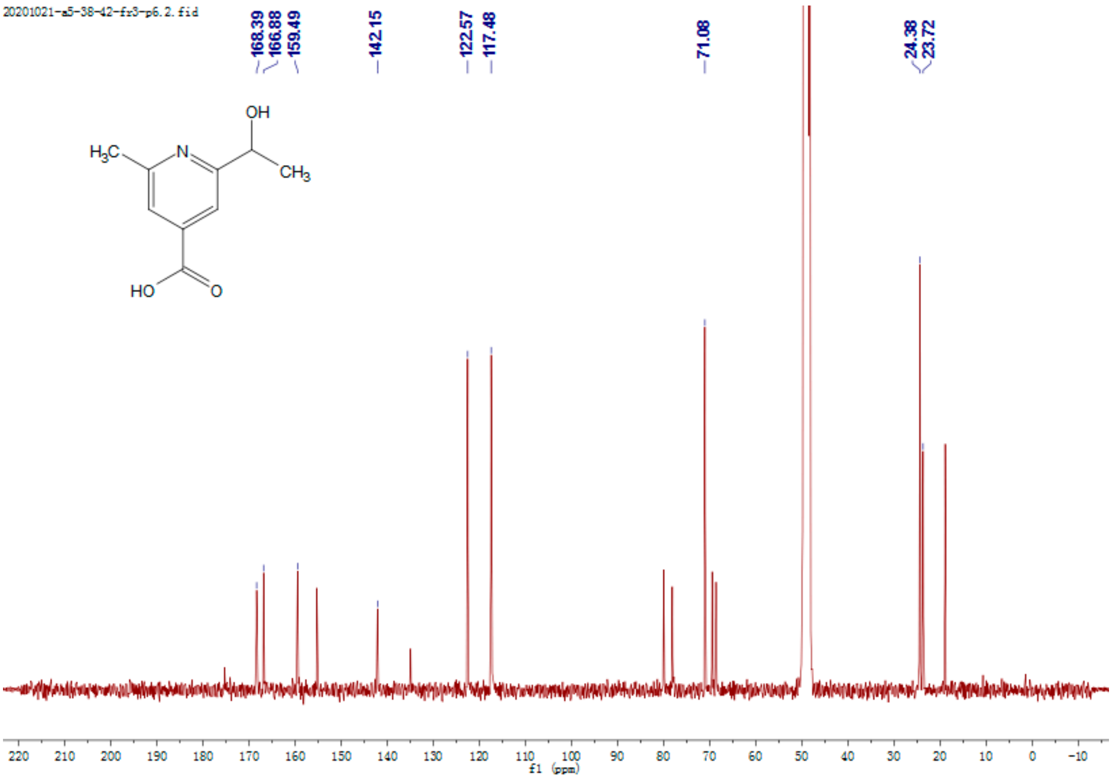

Figure S21. ^{13}C NMR spectrum of compound **7** in CD_3OD.

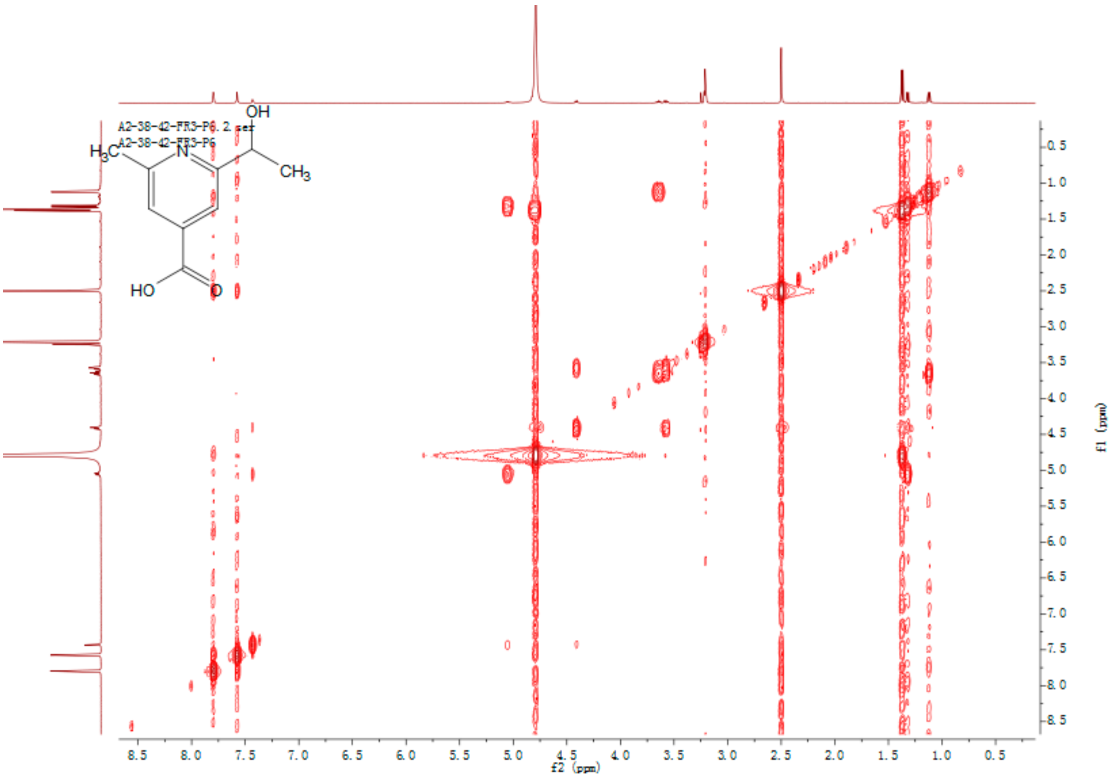

Figure S22. ^1H-^1H COSY spectrum of compound **7** in CD_3OD.

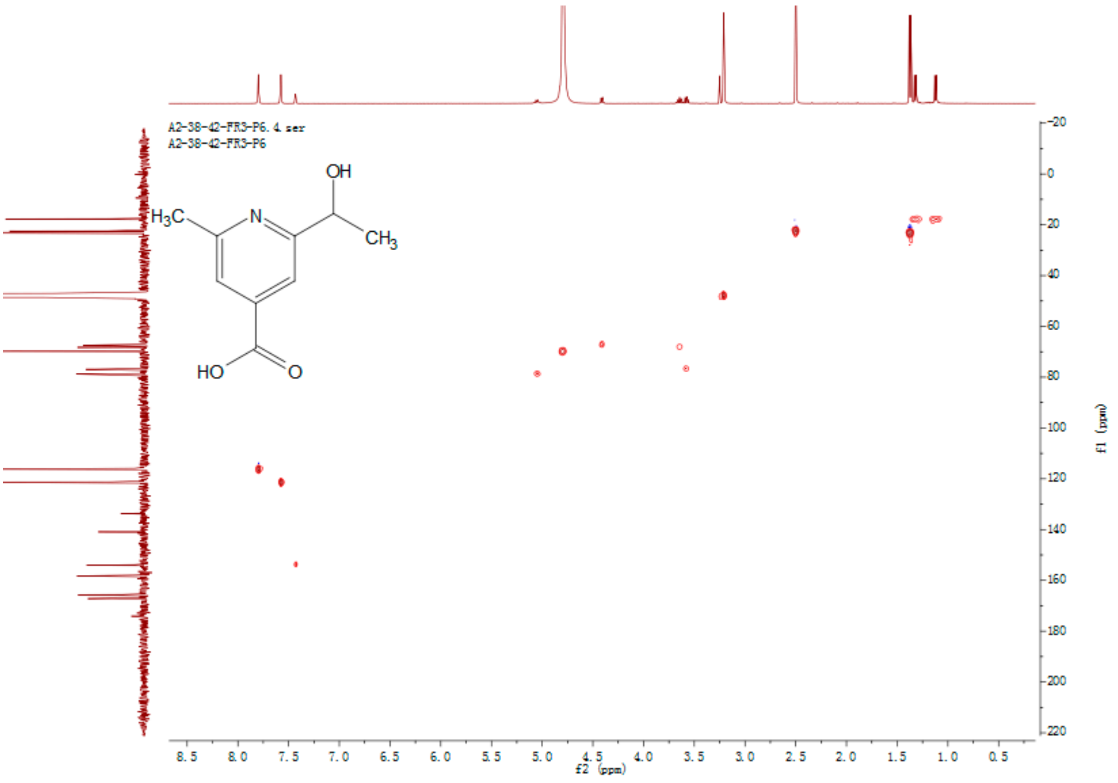

Figure S23. HSQC spectrum of compound **7** in CD_3OD.

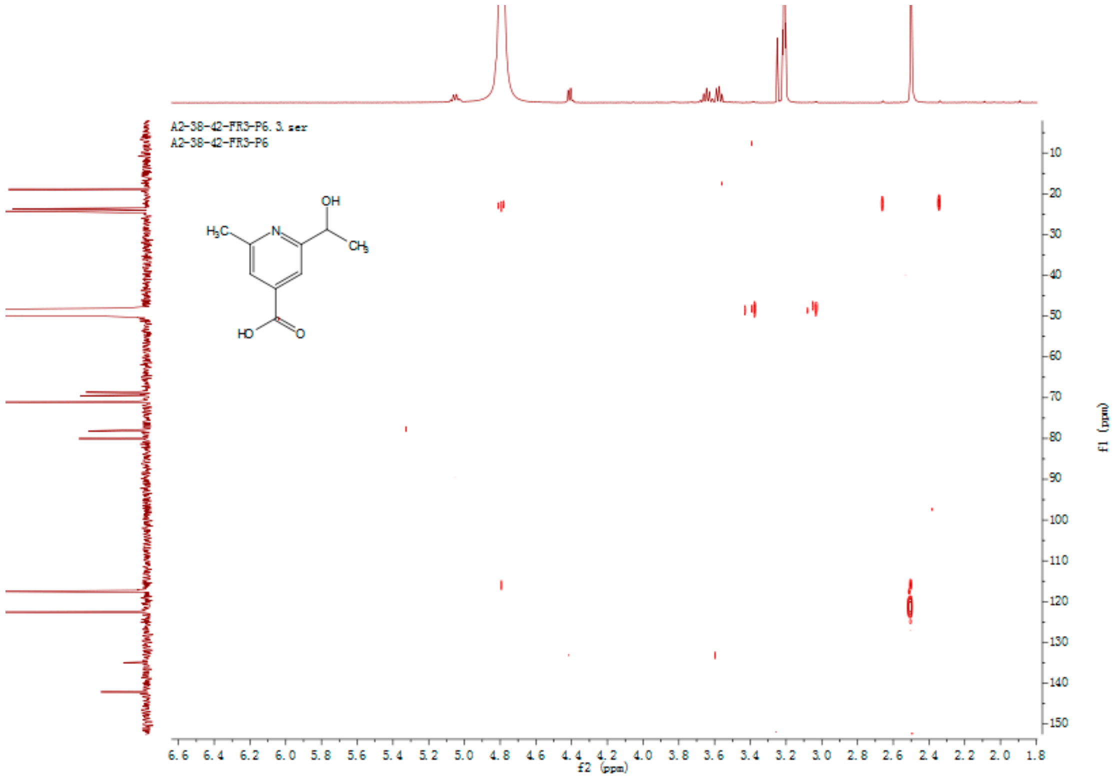

Figure S24. HMBC spectrum of compound **7** in CD_3OD.

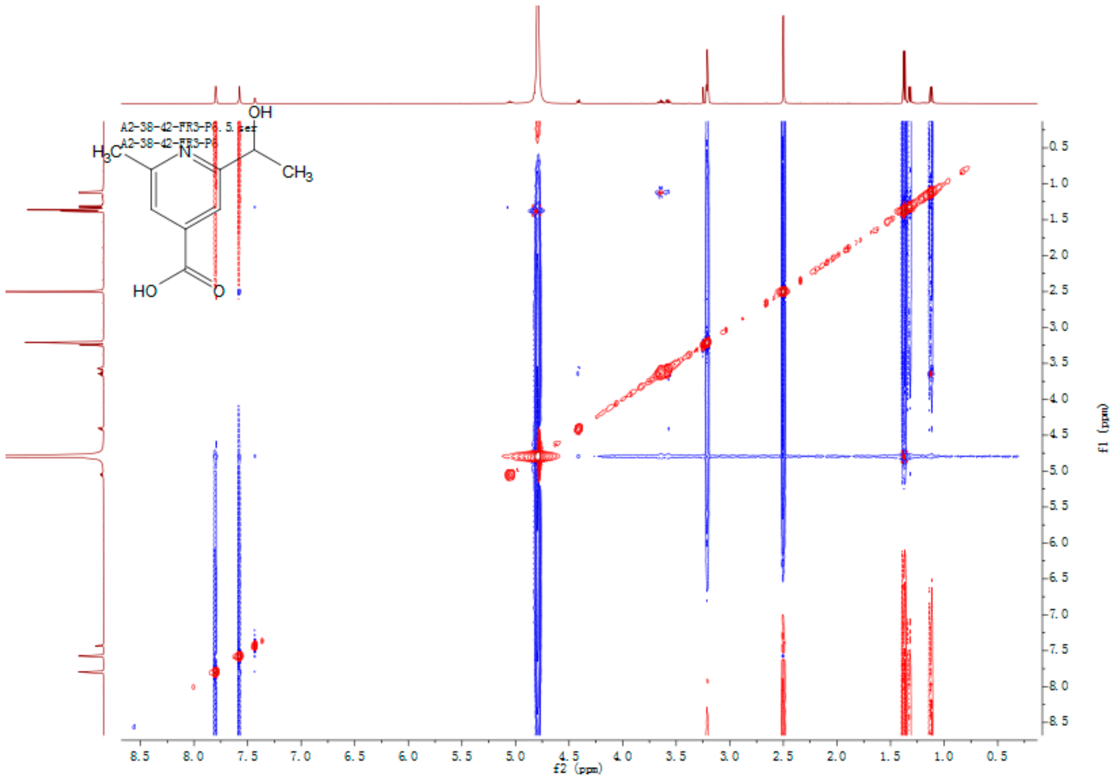

Figure S25. NOESY spectrum of compound **7** in CD_3OD.

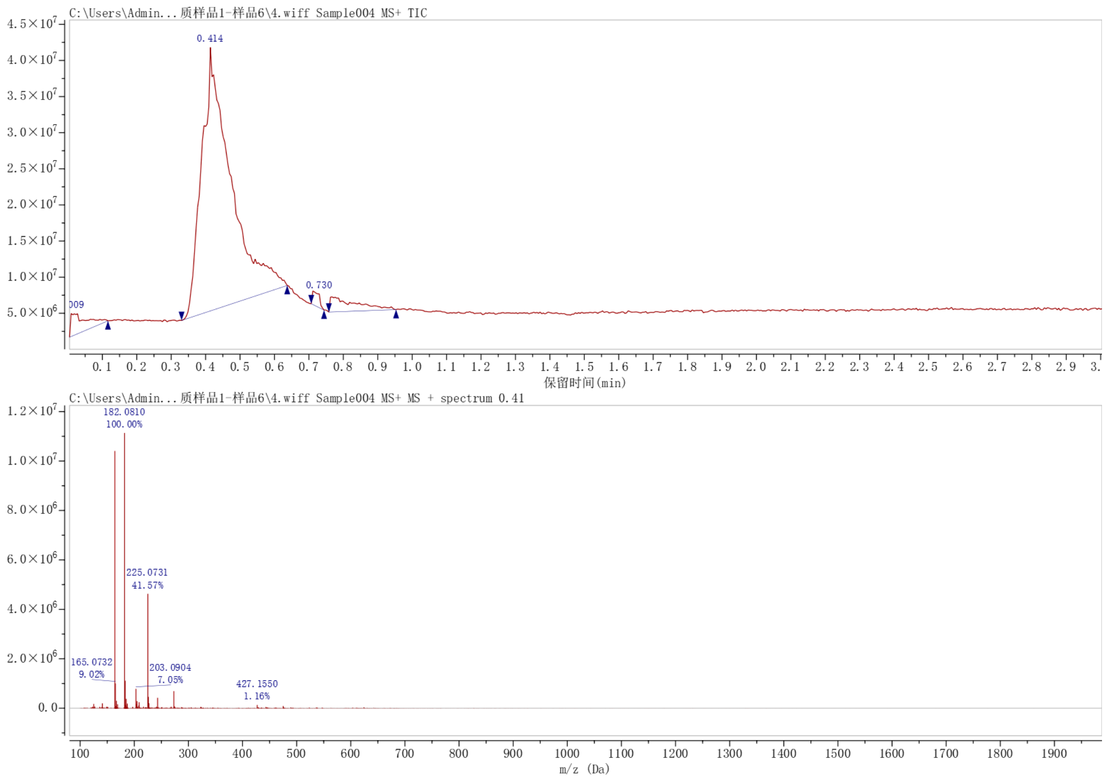

Figure S26. HRESIMS spectrum of compound **7.**

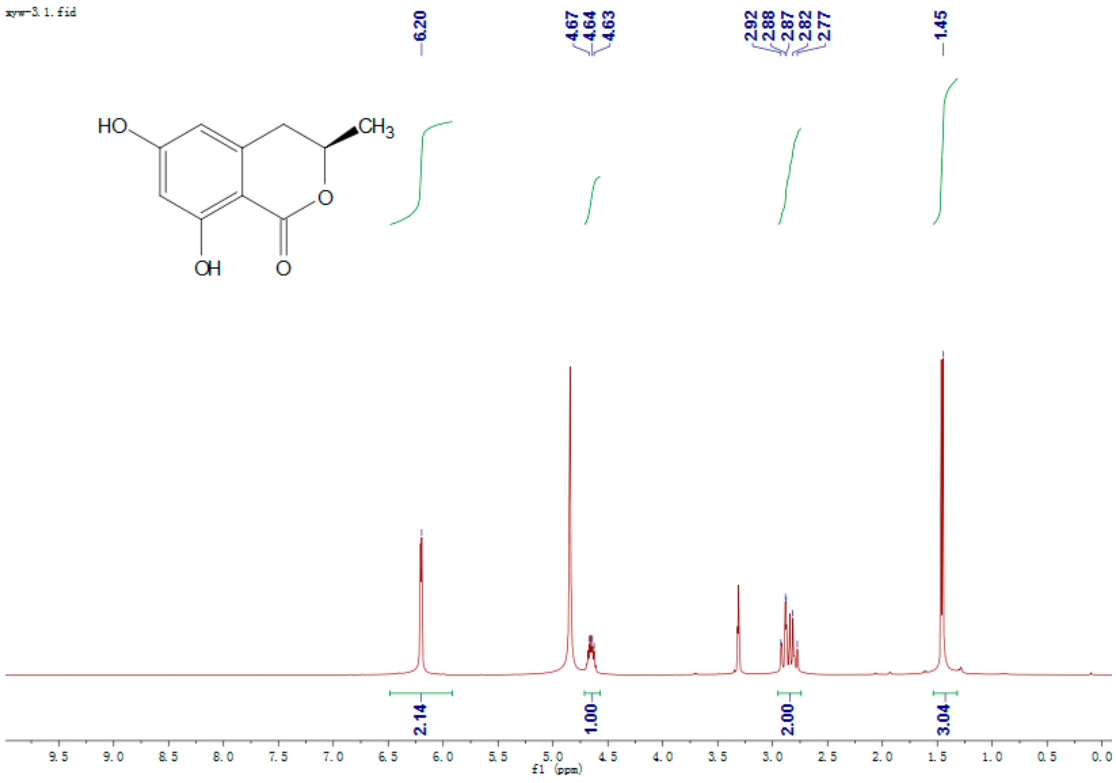

Figure S27. ^{1}H NMR spectrum of compound **8** in CD_3OD.

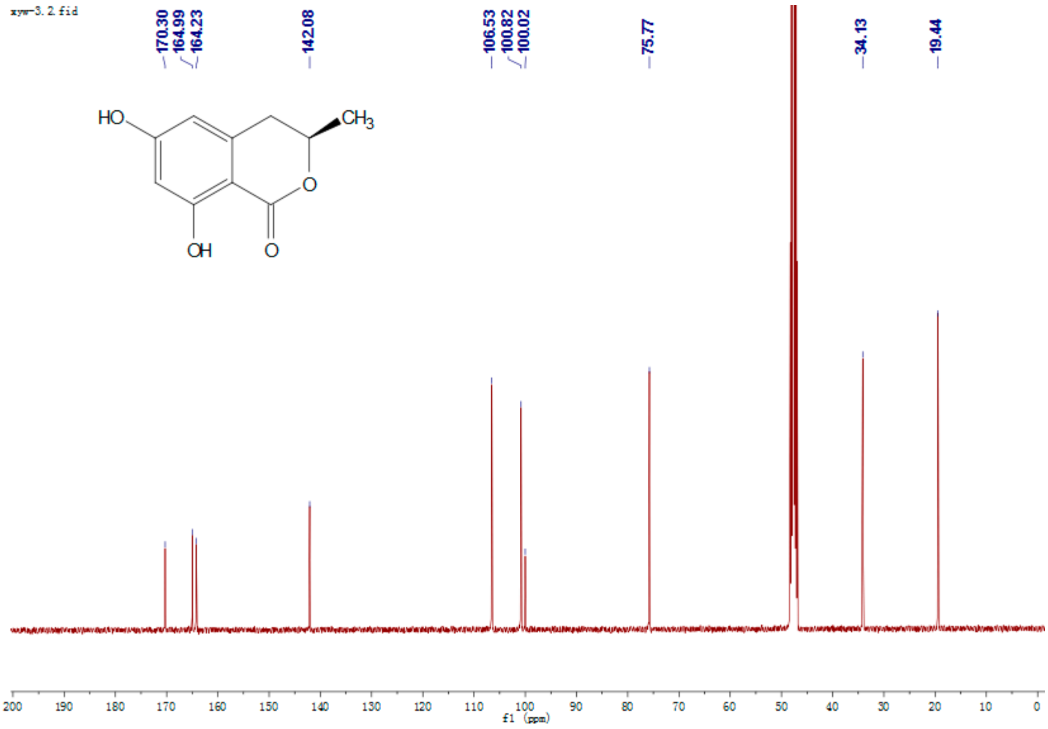

Figure S28. ^{13}C NMR spectrum of compound **8** in CD_3OD.

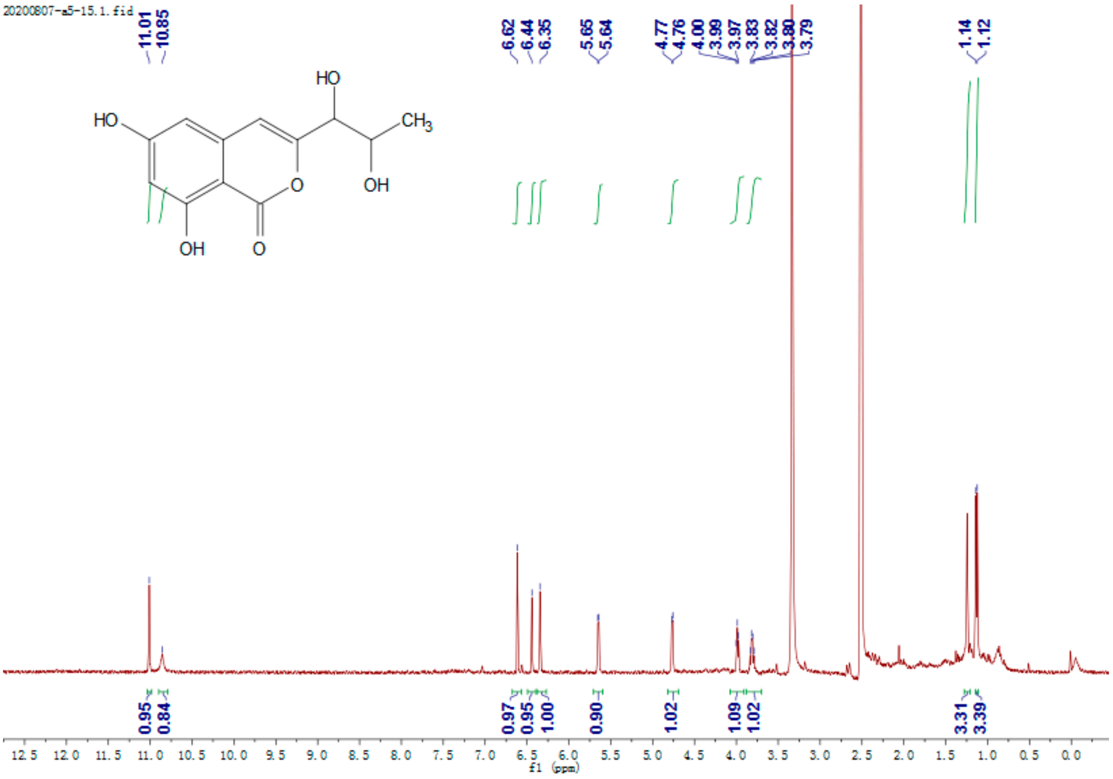

Figure S29. ^{1}H NMR spectrum of compound **9** in DMSO-d_6.

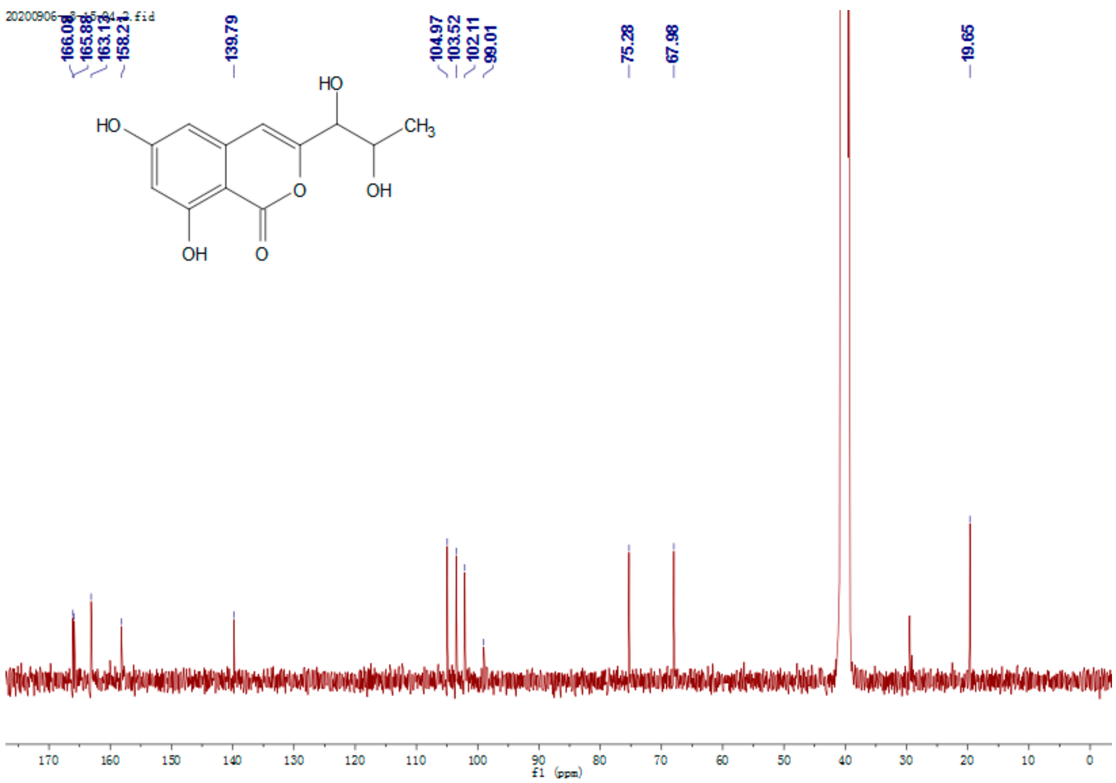

Figure S30. ^{13}C NMR spectrum of compound **9** in DMSO-d_6.

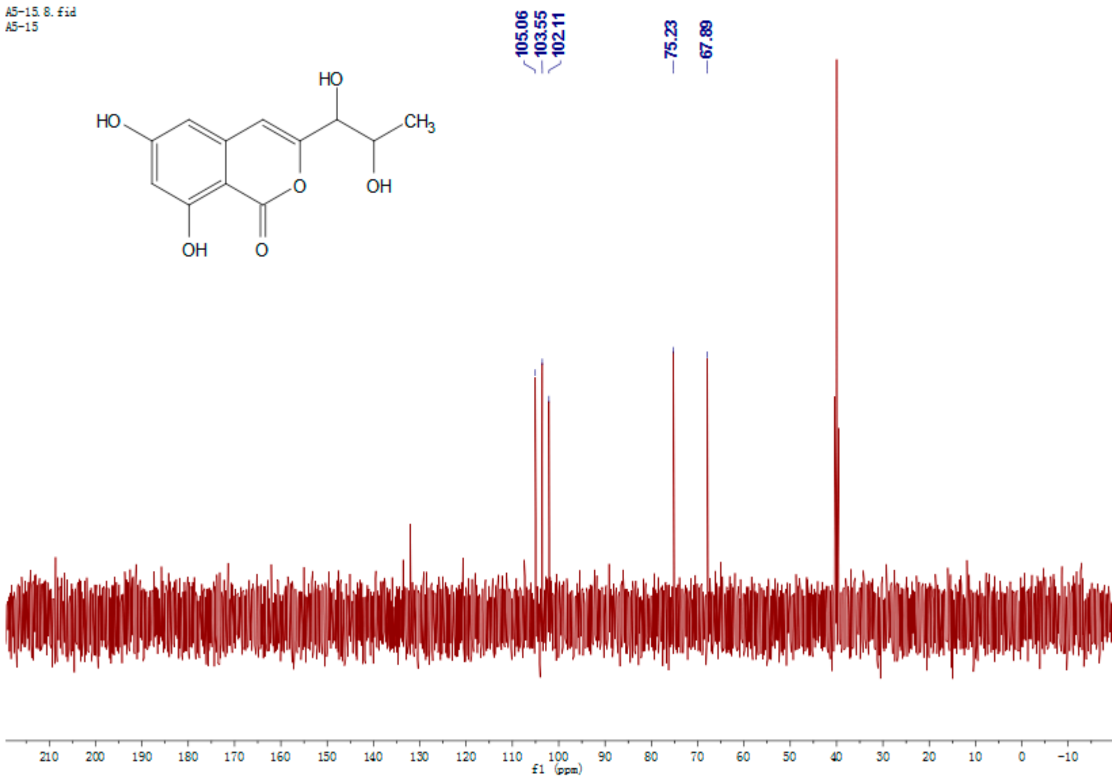

Figure S31. DEPT 90 spectrum of compound **9** in DMSO-d_6.

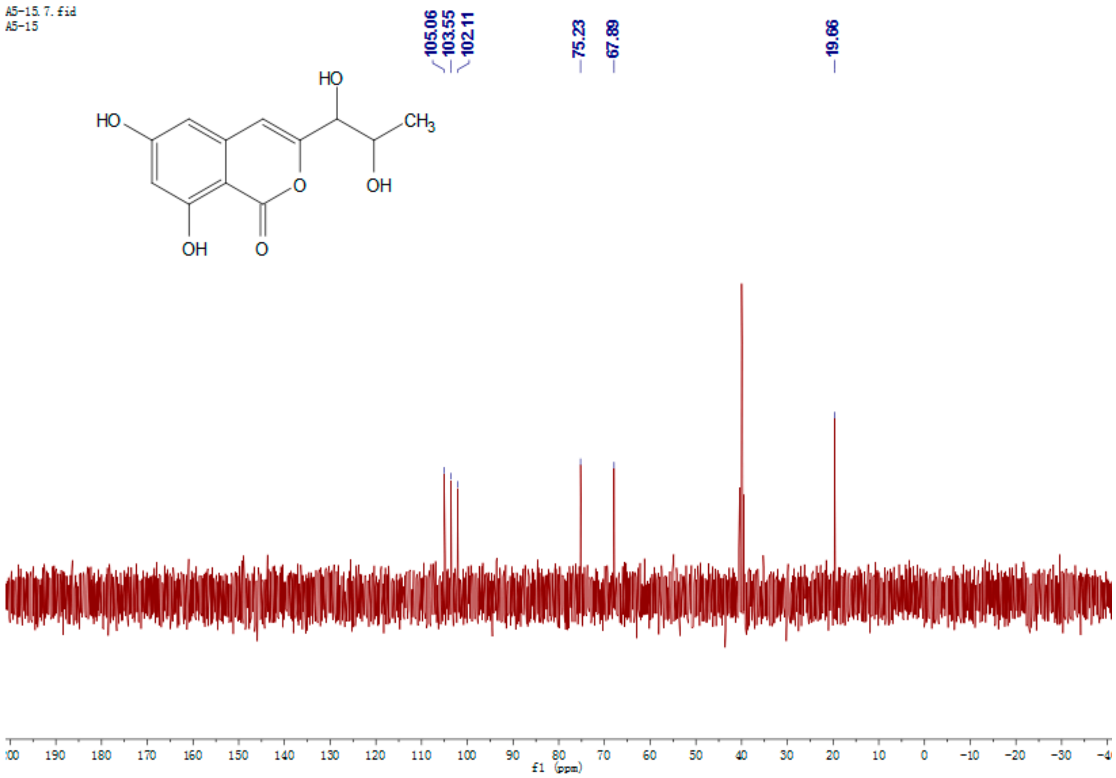

Figure S32. DEPT 135 spectrum of compound **9** in DMSO-d_6.

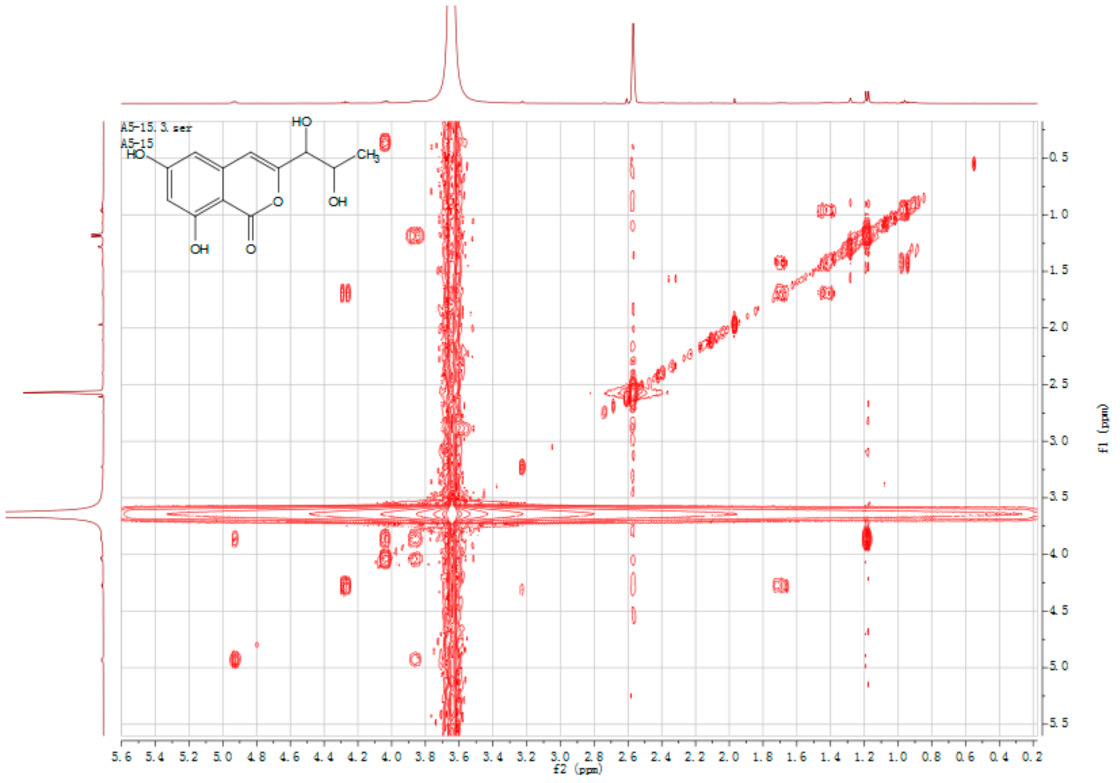

Figure S33. ^{1}H-^{1}H COSY spectrum of compound **9** in DMSO-d_6.

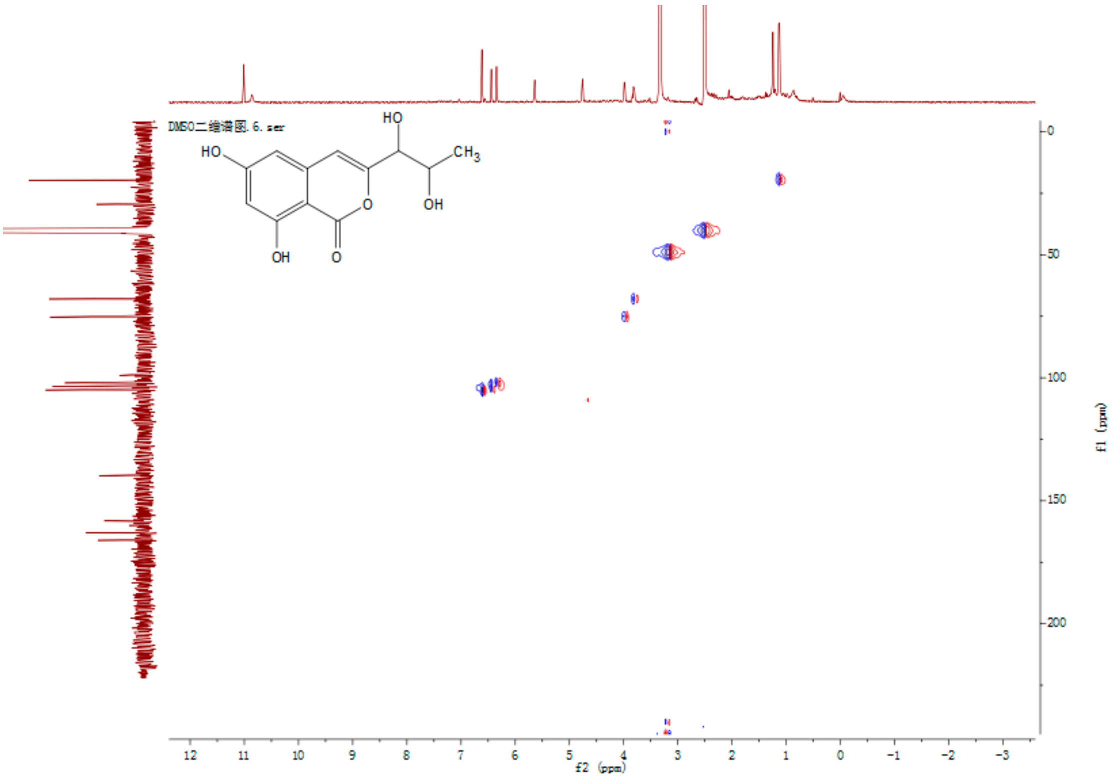

Figure S34. HSQC spectrum of compound **9** in DMSO-d_6.

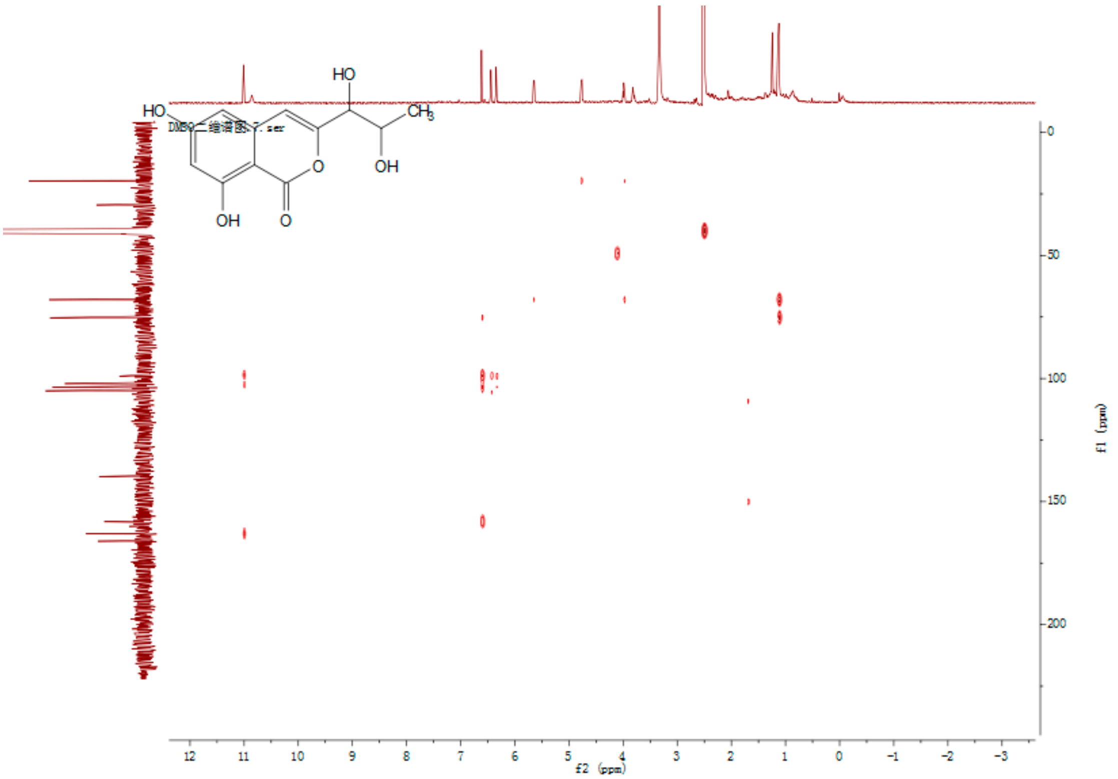

Figure S35. HMBC spectrum of compound **9** in DMSO-d_6.

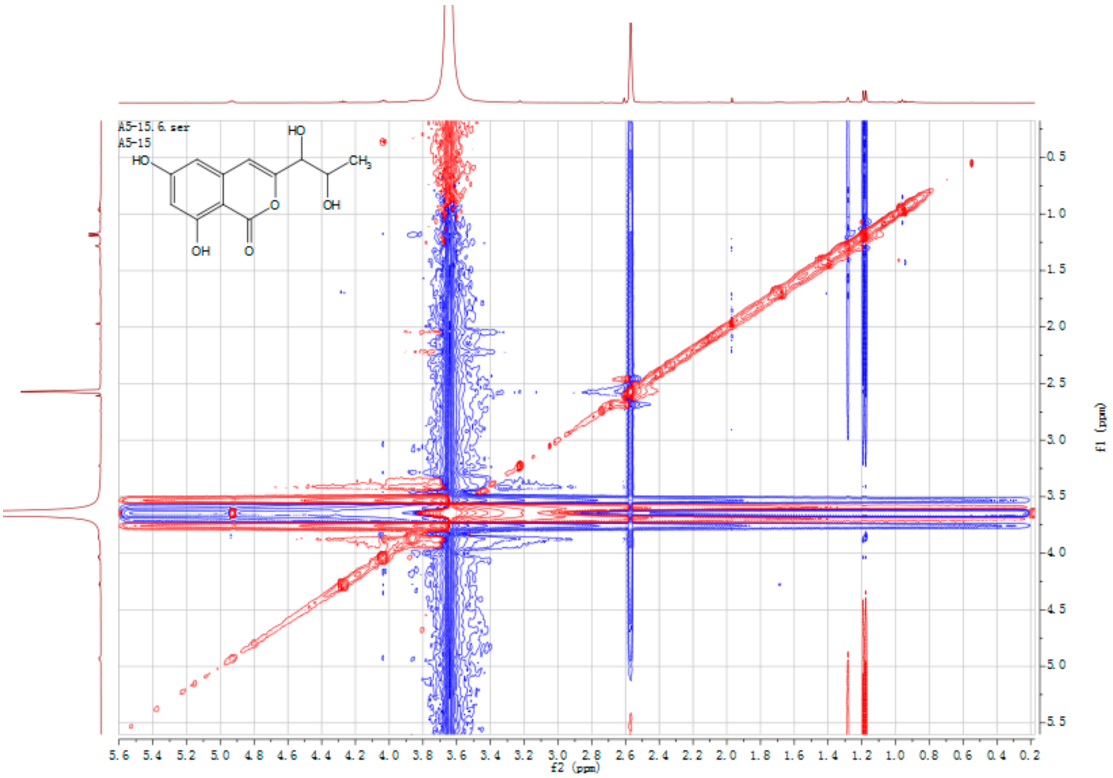

Figure S36. NOESY spectrum of compound **9** in DMSO-*d*$_6$.

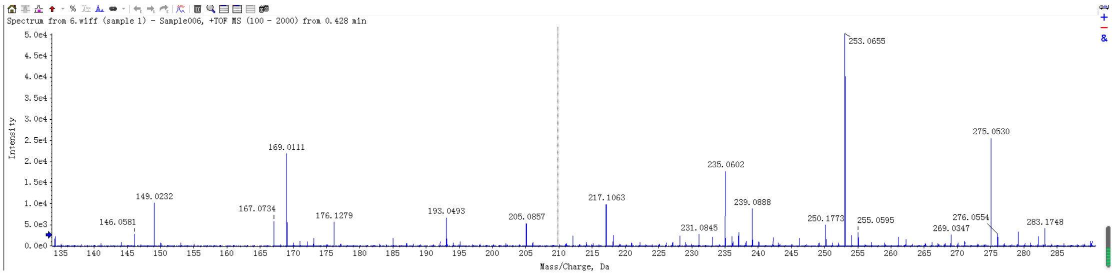

Figure S37. HRESIMS spectrum of compound **9.**

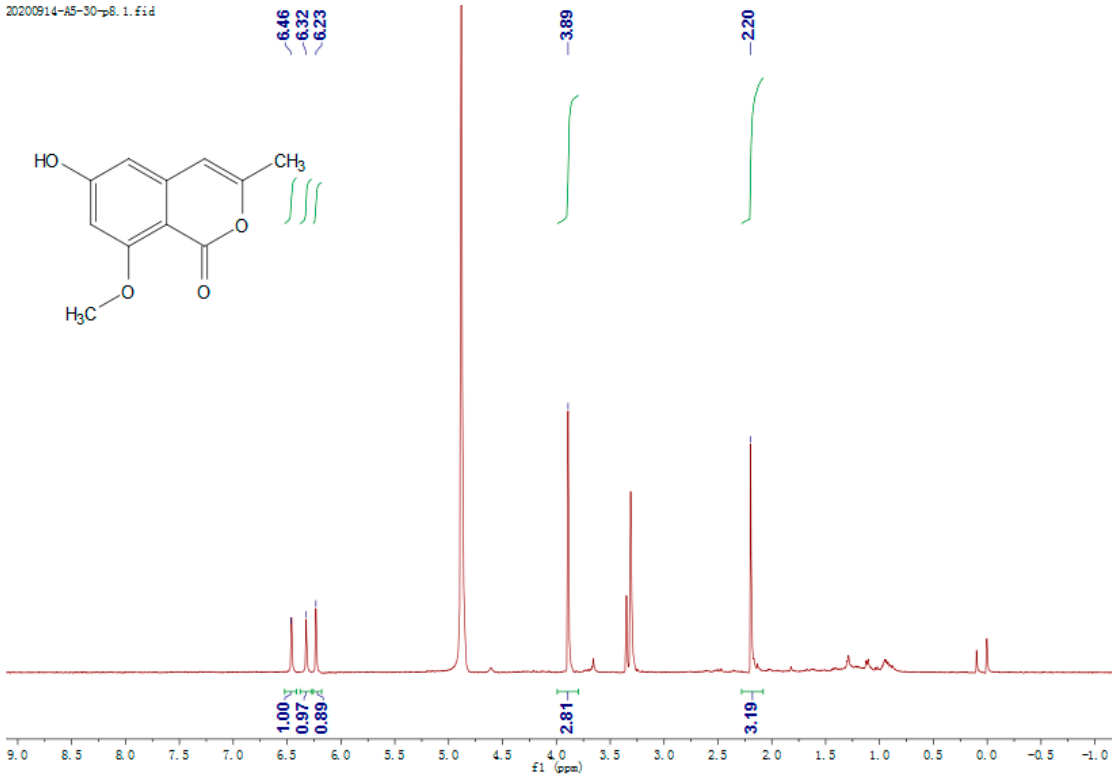

Figure S38. ^{1}H NMR spectrum of compound **10** in CD_3OD.

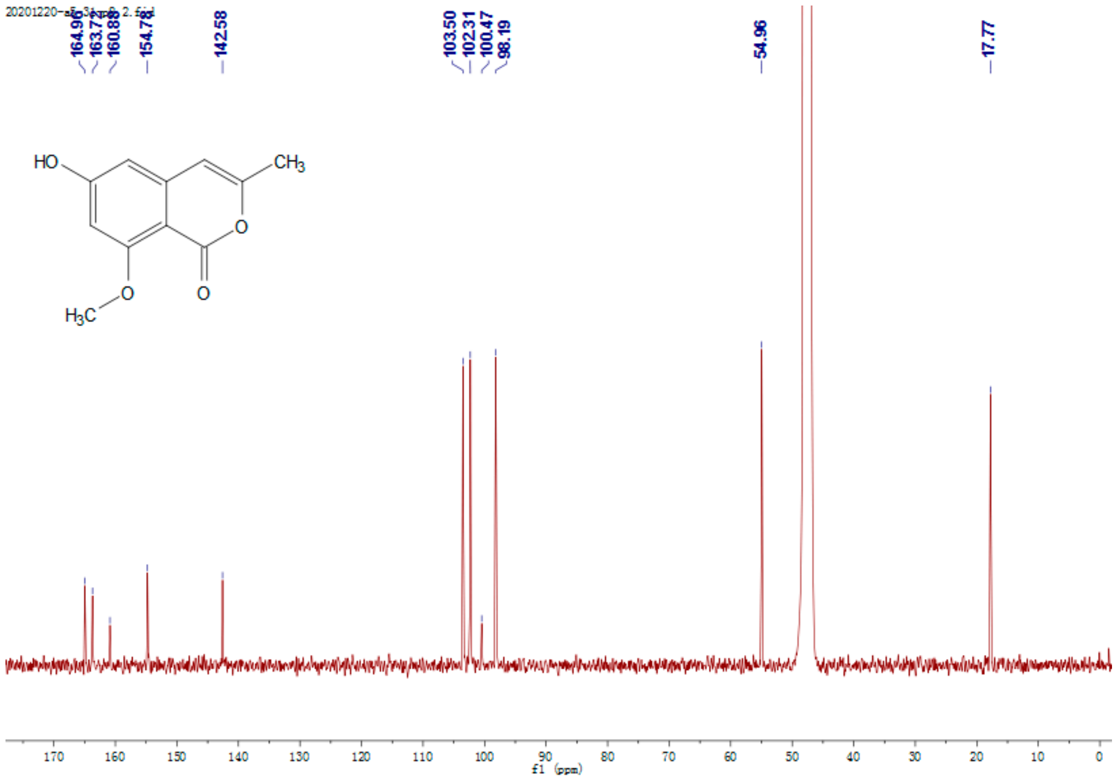

Figure S39. ^{13}C NMR spectrum of compound **10** in CD_3OD.

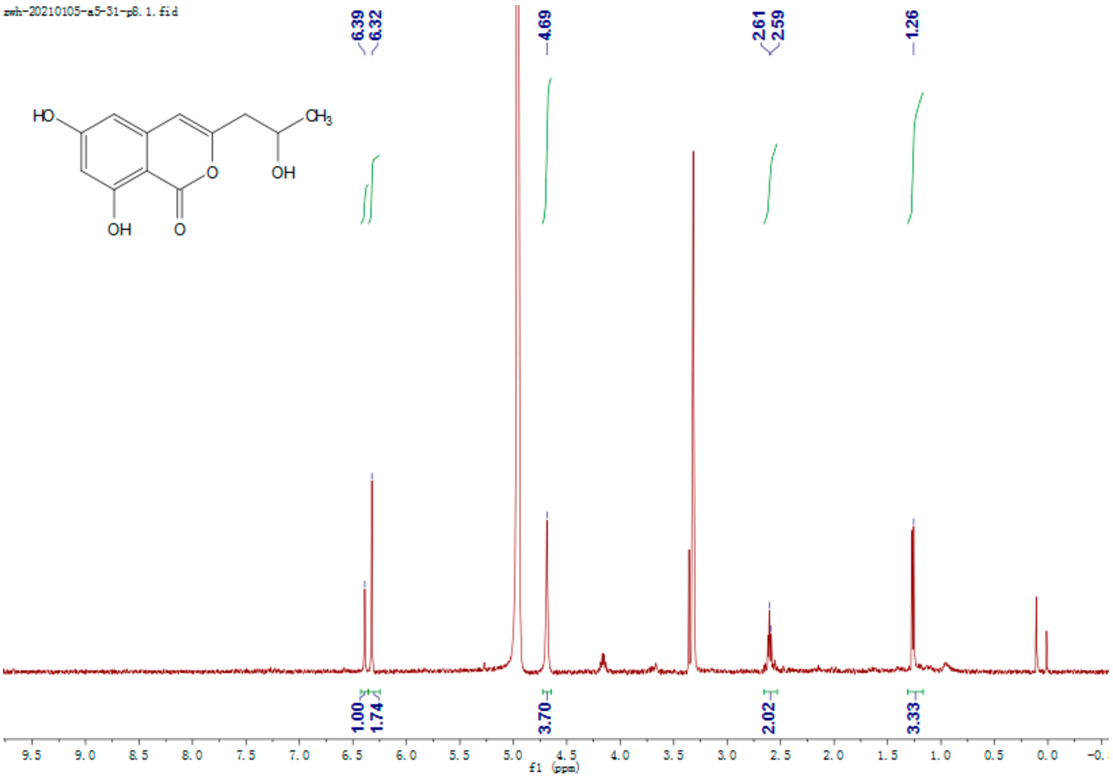

Figure S40. ^{1}H NMR spectrum of compound **11** in CD_3OD.

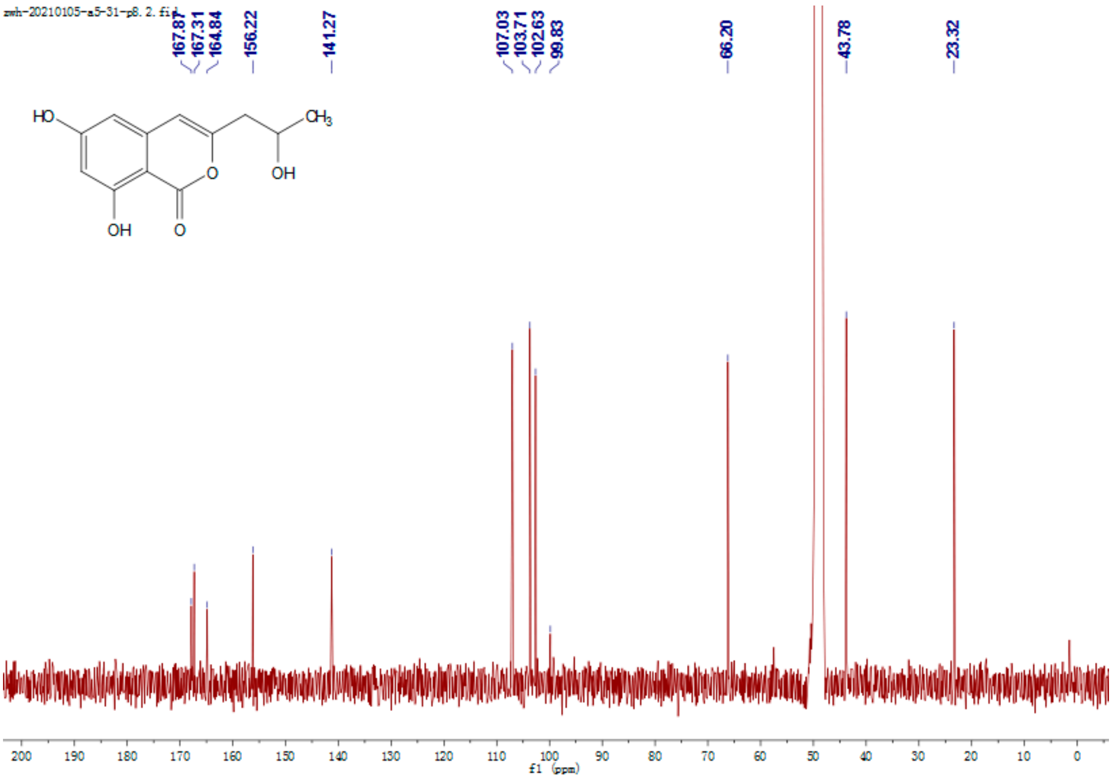

Figure S41. ^{13}C NMR spectrum of compound **11** in CD_3OD.
